# Changes in Circulating Stem and Progenitor Cell Numbers Following Acute Exercise in Healthy Human Subjects: a Systematic Review and Meta-analysis

**DOI:** 10.1007/s12015-020-10105-7

**Published:** 2021-01-02

**Authors:** M. Schmid, J. M. Kröpfl, C. M. Spengler

**Affiliations:** 1 Exercise Physiology Lab, Institute of Human Movement Sciences and Sport, ETH Zurich, Winterthurerstrasse 190, CH-8057 Zurich, Switzerland; 2 Zurich Center for Integrative Human Physiology (ZIHP), University of Zurich, Winterthurerstrasse 190, CH-8057 Zurich, Switzerland

**Keywords:** Stem and progenitor cells, Acute exercise, Meta-analysis, Systematic review, Stem cell mobilization, Endothelial, Hematopoietic, Mesenchymal, Kinetics, Moderator variables

## Abstract

Despite of the increasing number of investigations on the effects of acute exercise on circulating stem and progenitor cell (SC) numbers, and in particular on respective subgroups, i.e. endothelial (ESC), hematopoietic (HSC), and mesenchymal (MSC) stem and progenitor cells, a consensus regarding mechanisms and extent of these effects is still missing. The aim of this meta-analysis was to systematically evaluate the overall-effects of acute exercise on the different SC-subgroups and investigate possible subject- and intervention-dependent factors affecting the extent of SC-mobilization in healthy humans. Trials assessing SC numbers before and at least one timepoint after acute exercise, were identified in a systematic computerized search. Compared to baseline, numbers were significantly increased for early and non-specified SCs (enSCs) until up to 0.5 h after exercise (0–5 min: +0.64 [Standardized difference in means], *p* < 0.001; 6–20 min: +0.42, *p* < 0.001; 0.5 h: +0.29, *p* = 0.049), for ESCs until 12–48 h after exercise (0–5 min: +0.66, *p* < 0.001; 6–20 min: +0.43 *p* < 0.001; 0.5 h: +0.43, *p* = 0.002; 1 h: +0.58, *p* = 0.001; 2 h: +0.50, *p* = 0.002; 3–8 h: +0.70, *p* < 0.001; 12–48 h: +0.38, *p* = 0.003) and for HSCs at 0–5 min (+ 0.47, *p* < 0.001) and at 3 h after exercise (+ 0.68, *p* < 0.001). Sex, intensity and duration of the intervention had generally no influence. The extent and kinetics of the exercise-induced mobilization of SCs differ between SC-subpopulations. However, also definitions of SC-subpopulations are non-uniform. Therefore, finding a consensus with a clear definition of cell surface markers defining ESCs, HSCs and MSCs is a first prerequisite for understanding this important topic.

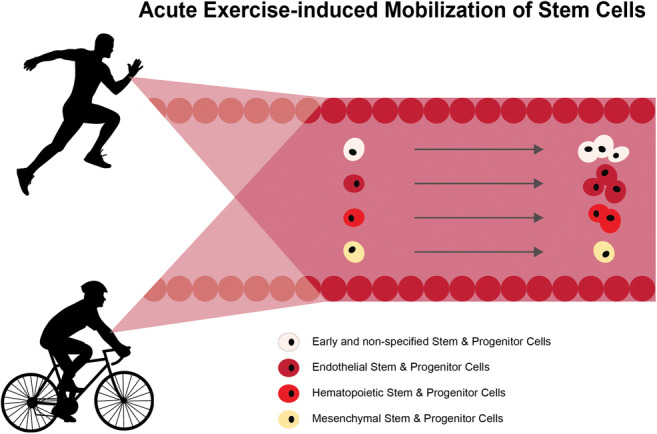

## Introduction

Stem cells (SCs) have the potential to self-renew and differentiate into cell types of specific tissues or organs, allowing for cell replacement and turnover. In adults, different groups of stem and progenitor cells, e.g. hematopoietic (HSCs), endothelial (ESCs), and mesenchymal stem and progenitor cells (MSCs) as well as very small embryonic-like stem cells [1] are found in circulation. These cells are able to differentiate into mature immune cells, endothelial cells, and cells of the connective tissue [2–4]. Given the physiologically important tasks of circulating stem and progenitor cells to repair and renew human tissue over the duration of an entire lifespan, and considering their promising application to treat a wide variety of degenerative conditions, research on potential stem cell mobilizing mechanisms is of great scientific interest [5].

Physical exercise as a non-invasive mechanism of adult stem and progenitor cell mobilization was first proposed around the 1980-ies with reports of increased hematopoietic stem and progenitor cell numbers (HSCs) measured in the peripheral blood after acute exercise [6–8]. An extensive and still growing body of literature has formed, reporting increased numbers not only of HSCs but also of circulating endothelial (ESCs) and mesenchymal (MSCs) stem and progenitor cells in the peripheral blood after physical activity [9–11]. However, several aspects of the study protocols make it difficult to understand mechanisms and kinetics. For example, exercise interventions differ substantially in type, intensity and duration, criteria for definition of the different cell subgroups as well as timepoints after exercise when cell numbers were assessed and also subject cohorts vary substantially between the different studies. Available reviews on this topic are either focused on a single cell population [12] and/or do not comply with current standards for systematic reviews [13–18]. Furthermore, to our knowledge, no comprehensive meta-analysis has yet been performed on this topic.

In the present study, we therefore conducted a comprehensive systematic review with meta-analyses on all available trials assessing numbers of early and non-specified adult stem and progenitor cells (enSCs) and respective SC-subgroups such as ESCs, HSCs, and MSCs in the peripheral blood, using flow cytometry or colony-forming-unit (CFU) assays before and at one or multiple timepoints after acute exercise. All reports conducted on healthy human subjects were included, regardless of the chosen study design. The main goal of the analysis was to assess whether and to which extent acute exercise mobilizes SCs and the different subgroups into the peripheral blood. As a secondary outcome, we aimed to identify possible moderating effects of sex, age, BMI and baseline physical activity level, as well as modality, duration, intensity and overall load of the exercise intervention.

## Methods

The present systematic review and meta-analysis is reported according to the PRISMA statement [19] and its corresponding explanation and elaboration [20].

### Review Protocol

The review question with the main and additional outcomes, as well as the strategy for the search, screening, extraction and analysis of the data were specified in advance. The review protocol can be made available upon request.

### Eligibility Criteria

No restrictions were imposed regarding the design of the studies as randomization/blinding or control conditions are neither possible nor feasible in the context of the investigated interventions. Further, no language or publication date restrictions were applied. Non-primary literature such as reviews, editorials, cross talks and journal clubs were excluded. Posters, (conference) abstracts and study outlines with no full text publication available were excluded if crucial information was missing.

All studies including healthy human subjects of any age, sex, ethnicity, baseline physical activity level and BMI were considered. In studies including patients, data of healthy control subjects were assessed where available. If two studies reported the same outcome of an overlapping or identical cohort of subjects, only the study of higher relevance was included.

Only studies assessing effects of acute exercise were included. However, studies implementing acute exercise in combination with blood flow restriction, hypoxia, nutritional supplementation of any kind and/or infusion of drugs or other compounds, without a control arm performing exercise-only, were excluded. Also, training studies were excluded except for data assessed in the context of acute exercise prior to any training intervention.

Included studies were required to report sufficient information on the primary outcome which included numbers of enSCs and/or respective subgroups, i.e. ESCs, HSCs, and/or MSCs, in the peripheral blood assessed via flow cytometry or colony forming-unit (CFU) assays before and at least once after acute exercise.

Secondary outcome measures included moderator variables like sex, age, BMI and baseline physical activity level of the study cohort, as well as duration, intensity, load and modality of the exercise intervention. These variables were assessed as comprehensively as possible but failure to report any secondary measure did not result in exclusion of a study.

If a study failed to report adequate statistical information, it was excluded.

### Information Sources

Electronic databases were screened via a computerized search of PubMed (MEDLINE database; 1966 to present), the Cochrane Library (1996 to present), EMBASE (1980 to present) and CINAHL (1961 to present) on 18 December 2018. A limited update search to identify newly published literature was performed in the form of 4 additional searches of the PubMed database between 18 and 2018 and 3 February 2020. In addition, citations and bibliographies of already positively qualified papers were hand searched.

### Search Strategy

The search strategy was kept general and aimed at identifying all studies investigating stem and progenitor cells before and after acute exercise. Stricter inclusion criteria were applied during the subsequent selection process. This approach was chosen in order to minimize the risk of missing relevant literature. Keywords and Medical Subject Heading (MeSH) terms were kept very general and included expressions such as “exercise”, “sport”, “physical activity” for the intervention domain and “stem cells”, “progenitor cells”, “circulating cells” for the primary outcome domain. The search was applied to “All fields” and terms were combined with the logical operator OR within a domain and the logical operator AND between the domains. The detailed search strategy for each database can be made available upon request. The resulting list of publications was cross-validated for completeness using a composition of randomly pre-selected references, all of which needed to be successfully identified by the applied search strategy.

### Study Selection

Eligibility was assessed independently by MS and JMK and consisted of a screening of the title, the abstract and the full-text, sequentially excluding records failing to meet the pre-defined eligibility criteria (see also section “Study Selection”). Disagreements between the two reviewers were resolved by consensus.

### Data Collection Process

A data extraction sheet was developed based on Harris et al., 2014 [21] and adapted to the primary and secondary outcome parameters at hand (see section “Data Items”). MS extracted the data of all included studies and JMK double checked the extracted data. Disagreements were resolved by discussion. If data points were accessible in graphical form only, MS and JMK independently extracted the data including error bounds using either the software PlotDigitizer version 2.6.8 [22] or by ruler and calculator. The average of the two separately extracted values (CV < 0.1%) was computed and used for the meta-analysis.

### Data Items

For each included publication, data was extracted on (1) general study information (author, journal, title, year of publication, year of patient enrollment, study design, country, possible conflicts of interest), (2) key statements (primary & secondary purposes, hypotheses), (3) subject cohort (in- & exclusion criteria, study rules, number of participants, sex, age, BMI, baseline physical activity level, smoking history, presence of a control group, medical history, ethnicity), (4) intervention (type/modality, duration, intensity, load, daytime of start, possible control interventions, several different interventions for the same participant group), (5) assessment of outcomes (procedure and timepoints of blood withdrawal, method of analysis (flow cytometry including the type of flow cytometer vs. CFU assay), method of cell isolation (lysis vs. density gradient centrifugation), cell surface marker combinations, cell estimates (relative number of cells present in % mononuclear cells or lymphocytes and/or absolute number of cells per µl blood or number of CFUs at all measured timepoints before/after the intervention or an estimate of the change of circulating cell numbers pre to post)), and (6) statistical tests performed.

### Risk of Bias within Studies

MS and JMK independently performed an individual risk of bias assessment for each included study, following a standardized checklist adapted from Moga et al., 2012 [23]. The checklist included 15 outcomes divided into the following categories: Study objective, Study population, Intervention, Outcome measures, Statistical analysis, Results and conclusion and Competing interests and source of support.

The assessment resulted in a quality score ranging from 0 to 15 points for each study. The final score for each study resulted from an average of the two separately assessed scores which did not differ more than a maximum of 3 score points. As none of the assessed studies yielded a score below 7.5 (50% of the maximum reachable score) no publication had to be excluded due to insufficient quality. The full quality assessment checklist can be made available upon request.

### Summary Measures

The measures extracted from the included studies were the absolute mean differences in cell numbers pre- to every measured timepoint post-exercise plus their standard deviations (SD) or the reported means of cell numbers pre- and post-intervention including their SD. If publications reported standard errors only (SE), SD was calculated using the following formula, where n = number of subjects:

1 $$SD = SE \times \sqrt{n}$$

Data reported as median and range was converted to mean ± SD using the approach proposed by Wan et al., 2014 [24].

The effect size for the assessment of the primary effect of acute exercise on circulating stem and progenitor cell numbers was the standardized difference in means (Std diff in means ± SE or Std diff in means ± 95% CI in the forest plots and analysis of bias across studies), as different studies reported the outcome in varying units. It was computed using the following formula:

2 $$Std diff = \frac{Paired diff}{\frac{{SD}_{Paired diff}}{\sqrt{2\times (1-r)}}}$$

where r = correlation coefficient and Paired diff = paired mean difference, which was either given or calculated via:

3 $$Paired diff = {mean}_{post}-{mean}_{pre}$$

and its respective standard deviation (SD_Paired diff_):

4 $${SD}_{Paired diff} = \sqrt{{(SD}_{pre}{)}^{2}+{(SD}_{post}{)}^{2}-2\times r\times {SD}_{pre}\times {SD}_{post}}$$

Secondary outcomes were moderator variables such as age and sex of the study population. A comprehensive list of the assessed parameters including their definition is provided in Table 1.

**Table 1:** Extracted moderator variables

Variable	Definition
Sex Age	Percentage of male participants Mean age of participants in years. If the range was reported, the mean of the range was used
BMI	Mean body mass index in kg·m^− 2^
Modality	Exercise modality (running, cycling, resistance or other)
Duration	Total exercise duration in minutes (with warm-up and cool-down not taken into account)
Intensity	Classified as 1 (low), 2 (moderate) or 3 (vigorous) according to Garber et al., 2011 [25]. If none of the exercise measures were reported, the following criteria additionally applied: (half- )marathon/ultra-distance races, all-out/time trials, incremental tests to exhaustion = 3; exercise at 100% and above the individual lactate/ventilatory or anaerobic threshold = 3, between 70% - 100% = 2; exercise between 0% - 40% of the individual peak power output/maximal work rate = 1, > 40% - 60% = 2, > 60% = 3.
Load	Product of duration$\times$intensity
Baseline physical activity level	Classified as active or sedentary. “sedentary” = participants were described as sedentary, participants engaged in less than 0.5 h of moderate activity 3 times/week [26], the reported VO_2max_ was below the age- and sex-specific average [27]. “active” = participants engaged in at least 0.5 h of moderate activity at least 3 times/week, the reported VO_2max_ was above the age- and sex-specific average.

### Statistical Analysis

According to cell surface marker combinations, assessed outcomes were grouped into 4 major stem and progenitor cell subgroups: early and non-specified (i.e. not further characterized) stem and progenitor cells (enSCs), endothelial stem and progenitor cells (ESCs), hematopoietic stem and progenitor cells (HSCs) and mesenchymal stem and progenitor cells (MSCs). Only cells that were specifically reported to be positive for a clear indicator such as CD45 for the hematopoietic lineage [28] or KDR for ESCs [29] were allocated to the respective group. Cell populations only reported to positively stain for general stem cell markers such as CD34 [30] or CD133 [31], were-called “non-specified” stem and progenitor cells and allocated to the enSC-group. Populations that were positive for CD34 or CD133 but still negative for any of the lineage specific markers were called “early stem and progenitor cells” and allocated to the enSC-group as well. Within these cell subgroups, the outcomes were again subdivided into bins reflecting the various timepoints of post-exercise assessments. A list of the classification of the different marker combinations into the 4 subgroups, as well as the respective timepoint bins formed within the groups is provided in Table 2. The subgroups and respective timepoint combinations were established in an attempt to minimize co-occurrences of studies in the same bins while maximizing numbers of outcomes clustered in a bin.

**Table 2:** Outcome classification into groups and bins

Cell Subgroup	Marker combinations/assays included	Timepoint bins
enSCs	CD34^+^ CD34^+^/CD33^−^ CD34^+^/CD38^−^ CD34^+^/CD45^−^ CD34^+^/CD45^−^/CD38^−^ CD34^+^/CD133^+^ CD34^+^/HLA-DR^−^ CD133^+^	0–5 min, 6–20 min, 0.5 h, 1 h, 2 h, 3–96 h
ESCs	CD34^+^/CD31^+^/KDR^+^ CD34^+^/CD4^−^/CD31^+^/CD133^+^ CD34^+^/CD45^−^/KDR^+^ CD34^+^/CD133^+^/KDR^+^ CD34^+^/CD133^−^/KDR^+^ CD34^+^/KDR^+^ CD34^+^/KDR^+^/CD133^+^/CD11b^−^ CD133^+^/KDR^+^ CD133^+^/VE-Cad^+^ CFU-ECs	0–5 min, 6–20 min, 0.5 h, 1 h, 2 h, 3–8 h, 12–48 h
HSCs	CD34^+^/CD33^+^ CD34^+^/CD38^+^ CD34^+^/CD45^+^ CD34^+^/HLA-DR^+^ CFU-GM	0–5 min, 6–20 min, 0.5 h, 1 h, 3 h, 12–24 h
MSCs	CD34^+^/CD45^−^/CD31^−^/CD105^+^ CD34^−^/CD45^−^/CD31^−^/CD105^+^ CD45^−^/CD29^+^/CD13^+^	0–5 min, 2 h

If a study (measuring the same subject group during the same intervention) reported outcomes for multiple timepoints within the same bin and/or employed different marker combinations belonging to the same subgroup, a combined effect across outcomes was computed according to Borenstein et al., 2010 [32].

If a study conducted more than one intervention on the same or an overlapping cohort of subjects, or if a cell population was assessed using two different approaches that were not mergeable (i.e. flow cytometry and CFU assay), the sample size was divided and all outcomes were still taken into account for the analysis. This approach was deemed the most suitable, as it adjusts the weight of the study while making sure that no data points are lost.

For each bin in every cell subgroup, a separate meta-analysis was performed. All analyses were conducted using the Comprehensive Meta-Analysis Version 3 software (Biostat, Englewood, NJ, USA) applying a random effects model with paired groups. Weight of single studies was assigned with the inverse variance method. As correlation coefficients (r) were not reported in the primary literature, we used r = 0.6, in regard of recommendations for imputing pre-to-post-correlations which vary between 0.5 [33] and 0.7 [34]. Heterogeneity was assessed using Chi-squared tests, *Q* and *I*^*2*^ statistics and statistical significance was set at *p* < 0.050.

### Risk of Bias across Studies

Publication bias due to studies reporting high effect sizes being more likely to be published and selective reporting within the published studies may affect the validity of the cumulative evidence [35]. We therefore assessed the risk of bias across studies in all analyses that included at least 20 effect sizes. In detail, the Std diff in means were plotted against their inversed standard errors, resulting in a funnel plot. Those plots were then tested for asymmetry using Egger’s regression test and a Begg and Mazumdar rank correlation (yielding Kendall’s tau, corrected for continuity). Additionally, Orwin’s *Fail - safe N* with the criterion for an effect size deemed “trivial” set at a Std diff in means of 0.05 and assuming an effect size of 0.00 for added studies, as well as Duval and Tweedies’ trim and fill method (looking for missing studies to the left of the mean in a random effects model) were applied.

### Sensitivity Analyses

In order to assess the magnitude of influence of every single effect size on the resulting cumulative effect size, one-study-removed analyses (or in the present context more properly termed one-effect-size-removed analyses) were performed. Additionally, the robustness of the obtained cumulative effect sizes was tested by running the analyses again while substituting the imputed correlation coefficient of 0.6 by 0.5 and 0.7. As a certain number of effect sizes are required for those tests to be meaningful, sensitivity analysis was conducted on all analyses calculated from at least 20 effect sizes.

### Moderator Variables

The influence of the different moderator variables on the obtained effect sizes was tested via subgroup analysis for categorical variables (i.e. modality and intensity of the intervention and subjects’ baseline physical activity level) or via meta-regression in the case of continuous, numerical variables (%male participants, age, BMI, duration and load of the intervention).

For subgroup analyses, the heterogeneity between effect sizes within a subgroup was computed using Q tests based on analysis of variance. A fully random effects model was used and study-to-study variance was assumed to be equal for all studies and was thus pooled across all studies.

Meta-regressions were performed by plotting the magnitude of the variable versus the magnitude of the effect size while using a separate model for each variable. Prediction capacity of the moderator was tested using a simultaneous test that all coefficients (excluding intercept) are zero.

Subject and intervention characteristics are reported as mean ± SD. All tests were pre-defined and statistical significance was set at *p* < 0.050. Analyses were performed whenever at least 20 effect sizes were available and the main analysis revealed a significant (*p* < 0.050) heterogeneity of at least *I*^*2*^ ≥ 50% among the included effect sizes.

## Results

### Study Selection

**Fig. 1:**
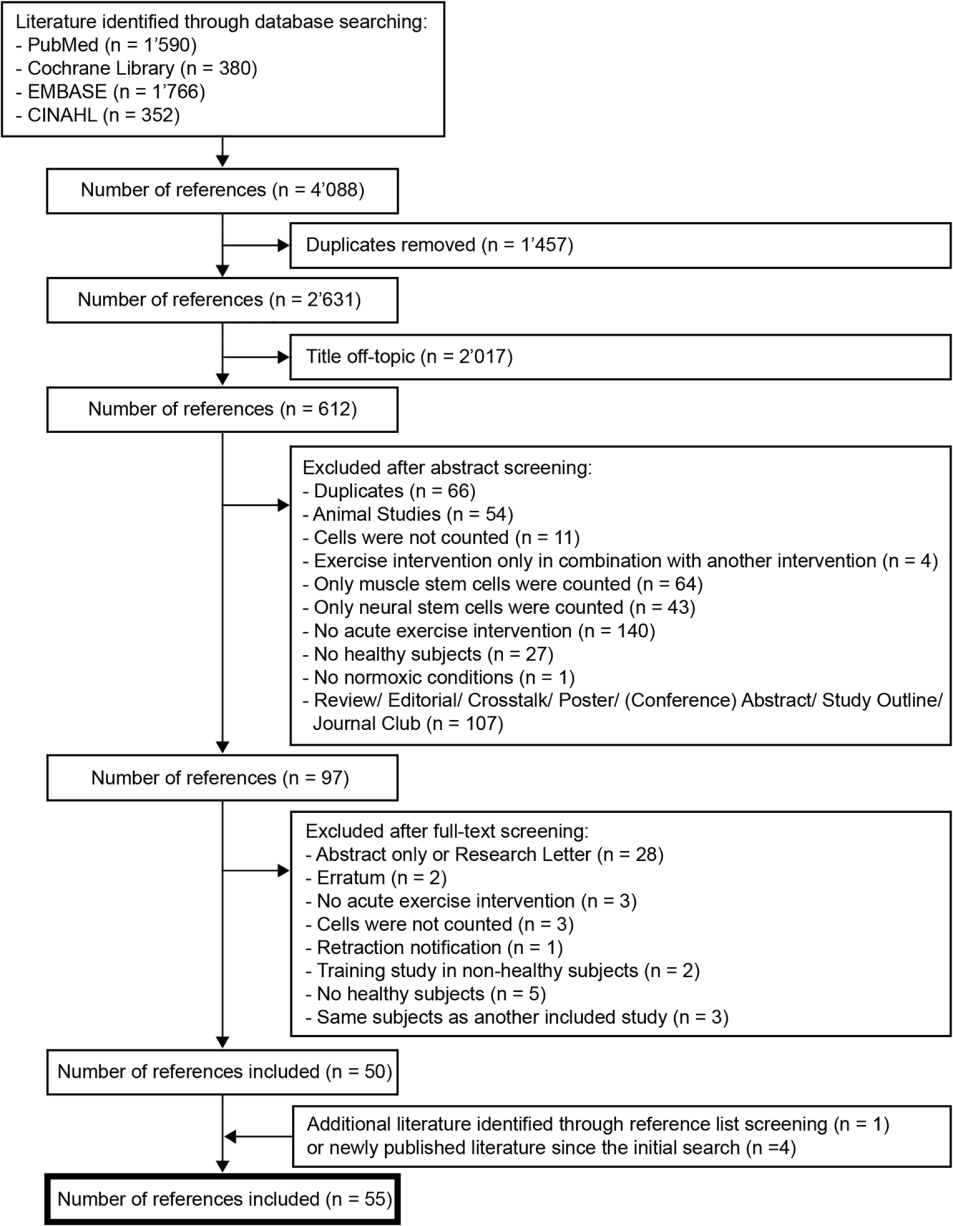
Flow diagram of the selection process

A total of 55 studies, resulting in 285 different effect sizes, were included in the review and meta-analysis. The initial search yielded 4088 records, of which 1457 were duplicates. Of the remaining 2631 records, 2017 were excluded due to their title being off-topic. 612 records were further considered and their abstracts screened for exclusion criteria. The remaining 97 publications were screened in full-text, where another 47 failed to meet the eligibility criteria and consequently were excluded. 50 studies were finally considered with another 5 studies additionally included in hindsight due to reference list screening of included papers and new publications being identified in the follow-up searches. Figure 1 depicts the selection process.

### Study Characteristics

A summary of extracted data for all included studies can be found in Table 3.

**Table 3:** Study characteristics

Reference	Intervention	Subject cohort	Sample size	Type of cells assessed
Adams et al., 2008 [36]	Marathon	Middle-aged, active Males	68	SCs, ESCs
Agha et al., 2018 [37], Part 1 (0.5 h)	0.5 h vigorous Running	Young, active Volunteers	15	SCs
Agha et al., 2018 [37], Part 1 (1.5 h)	1.5 h moderate Running	Young, active Volunteers	15	SCs
Agha et al., 2018 [37], Part 2	0.5 h vigrorous Cycling	Young Volunteers	12	SCs
Anz et al., 2019 [38]	20 min vigorous Cycling	Young Volunteers	20	SCs
Baker et al., 2017 [39] (30%)	40 min low-intensity Cycling	Young, active Males	11	SCs
Baker et al., 2017 [39] (70%)	18 min vigorous Cycling	Young, active Males	11	SCs
Barrett et al., 1978 [6]	4 min vigorous Stair Running	Young Volunteers	7	HSCs
Bonsignore et al., 2002 [40] (M)	Marathon	Middle-aged, active Males	8	SCs, HSCs
Bonsignore et al., 2002 [40] (HM)	Half-Marathon	Young, active Males	8	SCs, HSCs
Bonsignore et al., 2010 [41] (M)	Marathon	Middle-aged, active Males	9	SCs, ESCs, HSCs
Bonsignore et al., 2010 [41] (1.5 m)	1.5 km Time Trial Running	Middle-aged, active Males	8	SCs, ESCs, HSCs
Chang & Paterno et al., 2015 [42]	0.5 h vigorous Running	Young Males	5	ESCs
Cubbon et al., 2010 [43] (EU)	0.5 h moderate Cycling	Young, active Males	15	SCs, ESCs
Cubbon et al., 2010 [43] (SA)	0.5 h moderate Cycling	Young, sedentary Males	15	SCs, ESCs
Goussetis et al., 2009 [44]	Ultra-distance Running Race	Middle-aged Volunteers	10	ESCs
Harris et al., 2017 [45] (CONT)	0.5 h moderate Cycling	Middle-aged, sedentary Females	15	SCs, ESCs
Harris et al., 2017 [45] (MOD-INT)	0.5 h vigorous Cycling	Middle-aged, sedentary Females	15	SCs, ESCs
Harris et al., 2017 [45] (HIT)	0.5 h vigorous Cycling	Middle-aged, sedentary Females	9	SCs, ESCs
Heal & Brightman, 1987 [7] (stair)	Moderate Stair Running	Young Volunteers	10	HSCs
Heal & Brightman, 1987 [7] (cycling)	20 min moderate Cycling	Young Volunteers	5	HSCs
Jenkins et al., 2009 [46] (t)	0.5 h vigorous Running	Young, active Males	8	ESCs
Jenkins et al., 2009 [46] (s)	0.5 h vigorous Running	Young, sedentary Males	8	ESCs
Jootar et al., 1992 [8]	3.8 km Time Trial Running	Young Volunteers	19	HSCs
Kaźmierski et al., 2015 [47]	Incremental Treadmill Test to Exhaustion (Bruce)	Middle-aged Volunteers	33	ESCs
Kroepfl & Pekovits et al., 2012 [48]	Incremental Cycling Test to Exhaustion	Young, active Males	10	SCs, ESCs, HSCs
Krüger et al., 2015 [49] (CET)	Vigorous Cycling to Exhaustion	Young, sedentary Males	12	ESCs, HSCs
Krüger et al., 2015 [49] (RET)	Vigorous Resistance Exercise	Young Males	12	ESCs, HSCs
Krüger et al., 2015 [49] (ECC)	Vigorous, eccentric Running	Young, sedentary Males	12	ESCs, HSCs
Krüger et al., 2016 [50] (CONT)	0.5 h vigorous Cycling	Young, sedentary Males	23	ESCs, HSCs
Krüger et al., 2016 [50] (HIT)	5 × 3 min vigorous Cycling	Young, sedentary Males	23	ESCs, HSCs
Lansford et al., 2016 [51] (m)	Vigorous Cycling	Young, active Males	16	ESCs
Lansford et al., 2016 [51] (f)	Vigorous Cycling	Young, active Females	10	ESCs
Laufs & Urhausen et al., 2005 [9] (82%)	0.5 h vigorous Running	Young, active Males	25	SCs, ESCs
Laufs & Urhausen et al., 2005 [9] (0.5 h 68%)	0.5 h vigorous Running	Young, active Males	25	SCs, ESCs
Laufs & Urhausen et al., 2005 [9] (10 min 68%)	10 min vigorous Running	Young, active Males	10	SCs, ESCs
Lee et al., 2015 [52]	Vigorous Resistance Exercise	Young Males	9	SCs
Lockard et al., 2010 [53] (t)	0.5 h vigorous Cycling	Middle-aged, active Males	12	ESCs
Lockard et al., 2010 [53] (s)	0.5 h vigorous Cycling	Old, sedentary Males	11	ESCs
Lutz et al., 2016 [54]	0.5 h moderate Running	Young, sedentary Males	18	SCs, ESCs
Magalhães et al., 2020 [55]	8 × 1 min Vigorous Cycling	Young, active Males	9	SCs, ESCs
Möbius-Winkler & Hilberg et al., 2009 [56]	4 h moderate Cycling	Young, active Males	18	SCs, ESCs
Montgomery et al., 2019 [57]	Low-intensity Resistance Exercise	Young Males	9	SCs, ESCs
Morici et al., 2005 [58] (group 1)	1 km All-out Rowing	Young, active Volunteers	14	SCs
Morici et al., 2005 [58] (group 2)	1 km All-out Rowing	Young, active Volunteers	6	SCs
Niemiro et al., 2017 [59]	1 h vigorous Running	Young, active Males	7	SCs, ESCs, MSCs
Niemiro et al., 2018 [60]	25 km Time Trial in Wheelchair Athletes	Young, active Volunteers	8	SCs, ESCs, MSCs
O’Carroll et al., 2019 [61] (CONTEX)	45 min vigorous Cycling	Young Volunteers	12	SCs, ESCs
O’Carroll et al., 2019 [61] (SPRINT)	6 × 20sec vigorous Cycling	Young Volunteers	12	SCs, ESCs
Obeid et al., 2015 [62] (MICE)	2 × 0.5 h moderate Cycling	Active Children	5	ESCs
Obeid et al., 2015 [62] (HIIE)	6 × 4 × 15sec vigorous Cycling	Active Children	5	ESCs
Palange et al., 2006 [63]	20 min vigorous Cycling	Middle-aged, sedentary Volunteers	12	SCs, ESCs, HSCs
Ramírez et al., 2006 [11]	Half-Marathon	Young Volunteers	11	MSCs
Ribeiro et al., 2017 [64] (60%)	0.5 h moderate Resistance Exercise	Young, sedentary Females	13	ESCs
Ribeiro et al., 2017 [64] (70%)	0.5 h vigorous Resistance Exercise	Young, sedentary Females	12	ESCs
Ribeiro et al., 2017 [64] (80%)	0.5 h vigorous Resistance Exercise	Young, sedentary Females	13	ESCs
Rocha et al., 2015 [65]	40 min moderate Cycling	Young, sedentary Volunteers	9	ESCs
Ross et al., 2014 [66]	Low-intensity Resistance Exercise	Young, active Males	13	SCs, ESCs
Ross et al., 2017 [67] (young)	0.5 h vigorous Cycling	Young, active Males	8	SCs, ESCs
Ross et al., 2017 [67] (old)	0.5 h vigorous Cycling	Old, active Males	9	SCs, ESCs
Rummens et al., 2012 [68]	Incremental Cycling Test to Exhaustion	Middle-aged Volunteers	25	SCs, ESCs
Sapp et al., 2019 [69] (MOD)	0.5 h moderate Cycling	Young Males	9	SCs, ESCs
Sapp et al., 2019 [69] (HII)	0.5 h vigorous Cycling	Young Males	9	SCs
Shaffer et al., 2006 [10] (young)	Incremental Treadmill Test to Exhaustion (Bruce)	Young Volunteers	9	SCs, ESCs
Shaffer et al., 2006 [10] (old)	Incremental Treadmill Test to Exhaustion (Gardner)	Old Volunteers	13	SCs
Shill et al., 2016 [70] (m)	Incremental Treadmill Test to Exhaustion	Young, active Males	11	ESCs
Shill et al., 2016 [70] (f)	Incremental Treadmill Test to Exhaustion	Young, active Females	11	ESCs
Stelzer et al., 2015 [71]	Ultra-endurance Cycling Race	Young, active Volunteers	7	SCs, ESCs, HSCs
Strömberg et al., 2017 [72]	1 h moderate Cycling	Young, active Males	10	SCs, ESCs
Thijssen et al., 2006 [73] (ys)	Incremental Cycling Test to Exhaustion	Young, sedentary Males	8	SCs
Thijssen et al., 2006 [73] (yt)	Incremental Cycling Test to Exhaustion	Young, active Males	8	SCs
Thijssen et al., 2006 [73] (os)	Incremental Cycling Test to Exhaustion	Old, sedentary Males	8	SCs
Thorell et al., 2009 [74]	Vigorous Cycling Lesson	Young, active Volunteers	9	ESCs
Van Craenenbroeck et al., 2008 [75] (group 1)	Incremental Cycling Test to Exhaustion	Young, active Volunteers	11	SCs, ESCs
Van Craenenbroeck et al., 2008 [75] (group 2)	Incremental Cycling Test to Exhaustion	Young, active Volunteers	14	SCs, ESCs
Van Craenenbroeck et al., 2010 [76]	Incremental Cycling Test to Exhaustion	Middle-aged, active Volunteers	13	SCs, ESCs
Van Craenenbroeck et al., 2011 [77] (young)	Incremental Cycling Test to Exhaustion	Young, sedentary Males	4	SCs, ESCs
Van Craenenbroeck et al., 2011 [77] (old)	Incremental Cycling Test to Exhaustion	Old, sedentary Males	4	SCs, ESCs
Waclawovsky et al., 2016 [78] (AE)	40 min moderate Cycling	Young, sedentary Males	5	SCs, ESCs
Waclawovsky et al., 2016 [78] (RE)	40 min moderate Resistance Exercise	Young, sedentary Males	5	SCs, ESCs
Wang et al., 2014 [79] (group HT)	Incremental Cycling Test to Exhaustion	Young, sedentary Males	20	SCs, ESCs
Wang et al., 2014 [79] (group NT)	Incremental Cycling Test to Exhaustion	Young, sedentary Males	20	SCs, ESCs
Wardyn et al., 2008 [80]	Incremental Treadmill Test to Exhaustion (Bruce)	Young Volunteers	37	SCs
West et al., 2015 [81]	45 min vigorous Running	Young, active Males	9	ESCs
Witkowski et al., 2016 [82]	0.5 h vigorous Running	Young, active Males	9	ESCs
Yang et al., 2007 [83]	Incremental Treadmill Test to Exhaustion	Young Males	16	ESCs
Zaldivar et al., 2007 [84] (LP)	0.5 h vigorous Cycling	Late-pubertal, sedentary Boys	13	SCs
Zaldivar et al., 2007 [84] (EP)	0.5 h vigorous Cycling	Early-pubertal, sedentary Boys	14	SCs

SCs = general stem and progenitor cells, ESCs = endothelial stem and progenitor cells, HSCs = hematopoietic stem and progenitor cells, MSCs = mesenchymal stem and progenitor cells, HT = hypoxic training, NT = normoxic training, LP = late-pubertal, EP = early-pubertal, M = marathon, HM = half-marathon, AE = aerobic exercise, RE = resistance exercise, EU = european, SA = south-asian, ys = young sedentary, yt = young trained, os = old sedentary, t = trained, s = sedentary, m = male, f = female. Abbreviations written in big letters are names of interventions adapted from the respective studies

### Risk of Bias within Studies (Quality Assessment)

The individual risk of bias within each included study is shown in Table 4, which shows the total quality score assessed using the aforementioned standardized checklist. The average score (± SD) was 10.9 ± 1.3, with a range of 7.5–13.0. All studies reported their study objective, while only 80% sufficiently described the characteristics of the study cohort. 38% failed to impose explicit in- and exclusion criteria or the reporting thereof and none of the included studies recruited their participants consecutively while appropriately stating to do so. A clear and complete description of the study intervention was provided by 76% of studies. 98% clearly defined their outcome parameters, used appropriate methods to determine them and measured outcomes before and after the intervention. 98% further used adequate statistical tests to assess the relevant outcomes. All studies reported the length of follow up (latest timepoint of assessment) but only 18% reported the loss of follow-up. 91% provided comprehensive estimates of the random variability, while only 4% reported whether any adverse events had occurred. Conclusions of all studies were supported by the obtained results and 60% of the studies stated sources of funding and whether competing interests existed.

**Table 4:** Quality assessment

Reference	Quality Score (0–15)
Adams et al., 2008 [36]	11 ± 0.0
Agha et al., 2018 [37]	12.5 ± 0.7
Anz et al., 2019 [38]	9.5 ± 0.7
Baker et al., 2017 [39]	11.5 ± 0.7
Barrett et al., 1978 [6]	8 ± 0.0
Bonsignore et al., 2002 [40]	11.5 ± 2.1
Bonsignore et al., 2010 [41]	9.5 ± 0.7
Chang & Paterno et al., 2015 [42]	10 ± 0.0
Cubbon et al., 2010 [43]	11 ± 1.4
Goussetis et al., 2009 [44]	10.5 ± 2.1
Harris et al., 2017 [45]	11.5 ± 2.1
Heal & Brightman, 1987 [7]	9 ± 0.0
Jenkins et al., 2009 [46]	10.5 ± 0.7
Jootar et al., 1992 [8]	7.5 ± 2.1
Kaźmierski et al., 2015 [47]	11 ± 1.4
Kroepfl & Pekovits et al., 2012 [48]	12 ± 0.0
Krüger et al., 2015 [49]	10.5 ± 0.7
Krüger et al., 2016 [50]	12 ± 0.0
Lansford et al., 2016 [51]	12 ± 0.0
Laufs & Urhausen et al., 2005 [9]	10.5 ± 0.7
Lee et al., 2015 [52]	12.5 ± 0.7
Lockard et al., 2010 [53]	12.5 ± 0.7
Lutz et al., 2016 [54]	12 ± 0.0
Magalhães et al., 2020 [55]	13 ± 0.0
Möbius-Winkler & Hilberg et al., 2009 [56]	10.5 ± 2.1
Montgomery et al., 2019 [57]	12 ± 0.0
Morici et al., 2005 [58]	11.5 ± 0.7
Niemiro et al., 2017 [59]	12.5 ± 0.0
Niemiro et al., 2018 [60]	12 ± 2.1
O’Carroll et al., 2019 [61]	10 ± 0.0
Obeid et al., 2015 [62]	11.5 ± 0.7
Palange et al., 2006 [63]	10 ± 0.0
Ramírez et al., 2006 [11]	9.5 ± 0.7
Ribeiro et al., 2017 [64]	13 ± 0.0
Rocha et al., 2015 [65]	11 ± 0.0
Ross et al., 2014 [66]	11 ± 0.7
Ross et al., 2017 [67]	10.5 ± 0.0
Rummens et al., 2012 [68]	11 ± 0.0
Sapp et al., 2019 [69]	13 ± 0.0
Shaffer et al., 2006 [10]	8 ± 1.4
Shill et al., 2016 [70]	11.5 ± 0.7
Stelzer et al., 2015 [71]	12 ± 1.4
Strömberg et al., 2017 [72]	11.5 ± 0.7
Thijssen et al., 2006 [73]	10.5 ± 0.7
Thorell et al., 2009 [74]	10.5 ± 0.7
Van Craenenbroeck et al., 2008 [75]	11 ± 0.0
Van Craenenbroeck et al., 2010 [76]	10 ± 0.0
Van Craenenbroeck et al., 2011 [77]	10 ± 0.0
Waclawovsky et al., 2016 [78]	11.5 ± 0.7
Wang et al., 2014 [79]	12 ± 1.4
Wardyn et al., 2008 [80]	8 ± 0.0
West et al., 2015 [81]	13 ± 1.4
Witkowski et al., 2016 [82]	11.5 ± 0.7
Yang et al., 2007 [83]	10 ± 0.0
Zaldivar et al., 2007 [84]	11 ± 0.0

Quality score is displayed as mean ± SD

### Results of Individual Studies

Following, forest plots and individual results of meta-analyses for all timepoints/bins within the assessed stem and progenitor cell populations are reported. Forest plots depict effect sizes which are Std diff in means (95% CI), calculated from cell numbers measured before and at/within the indicated timepoint/-span after exercise. The weight of each effect size is represented by the size of its corresponding symbol. The combined data is always shown in the last row.

### Early and Non-specified Stem and Progenitor Cells (enSCs)

**Fig. 2:**
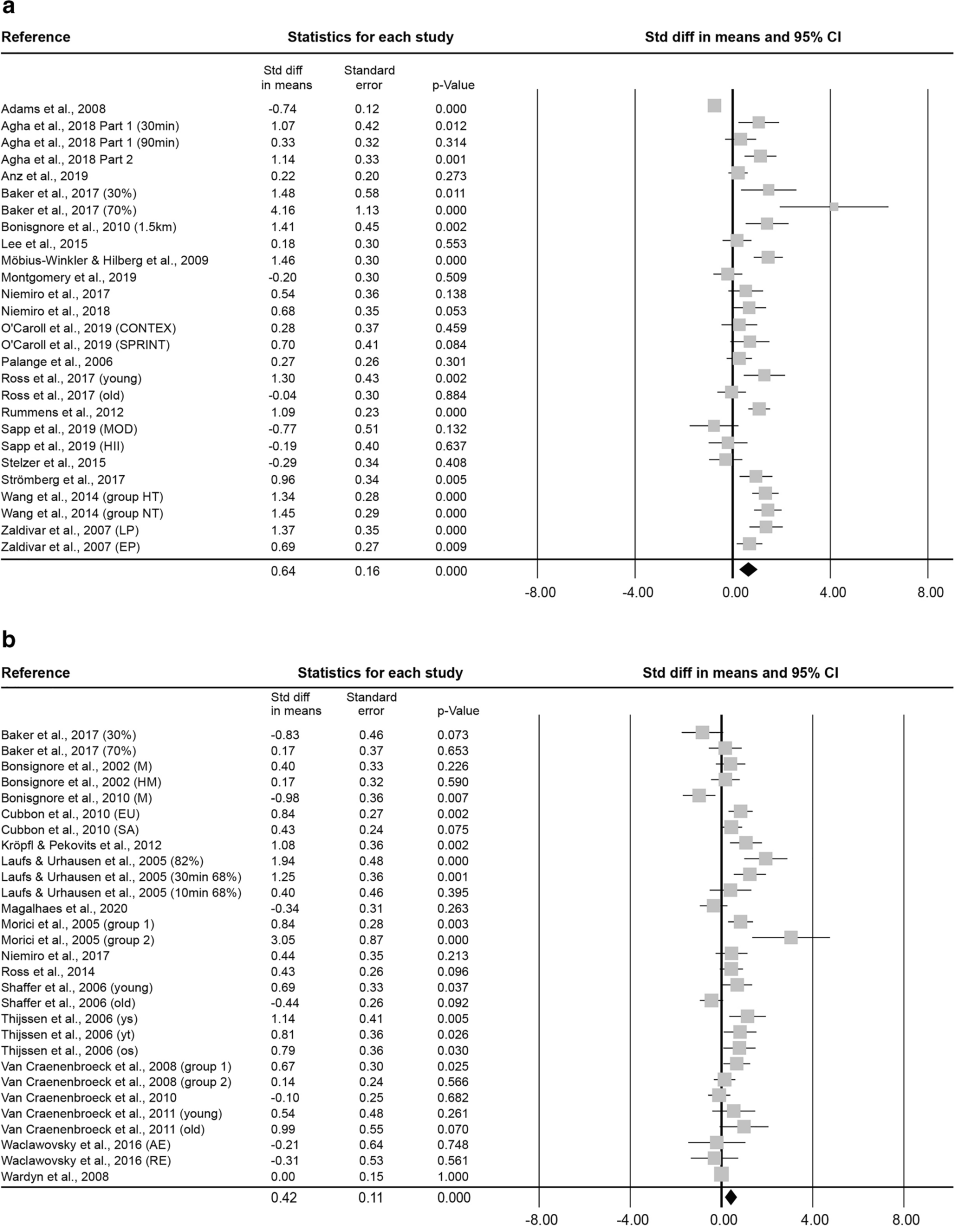

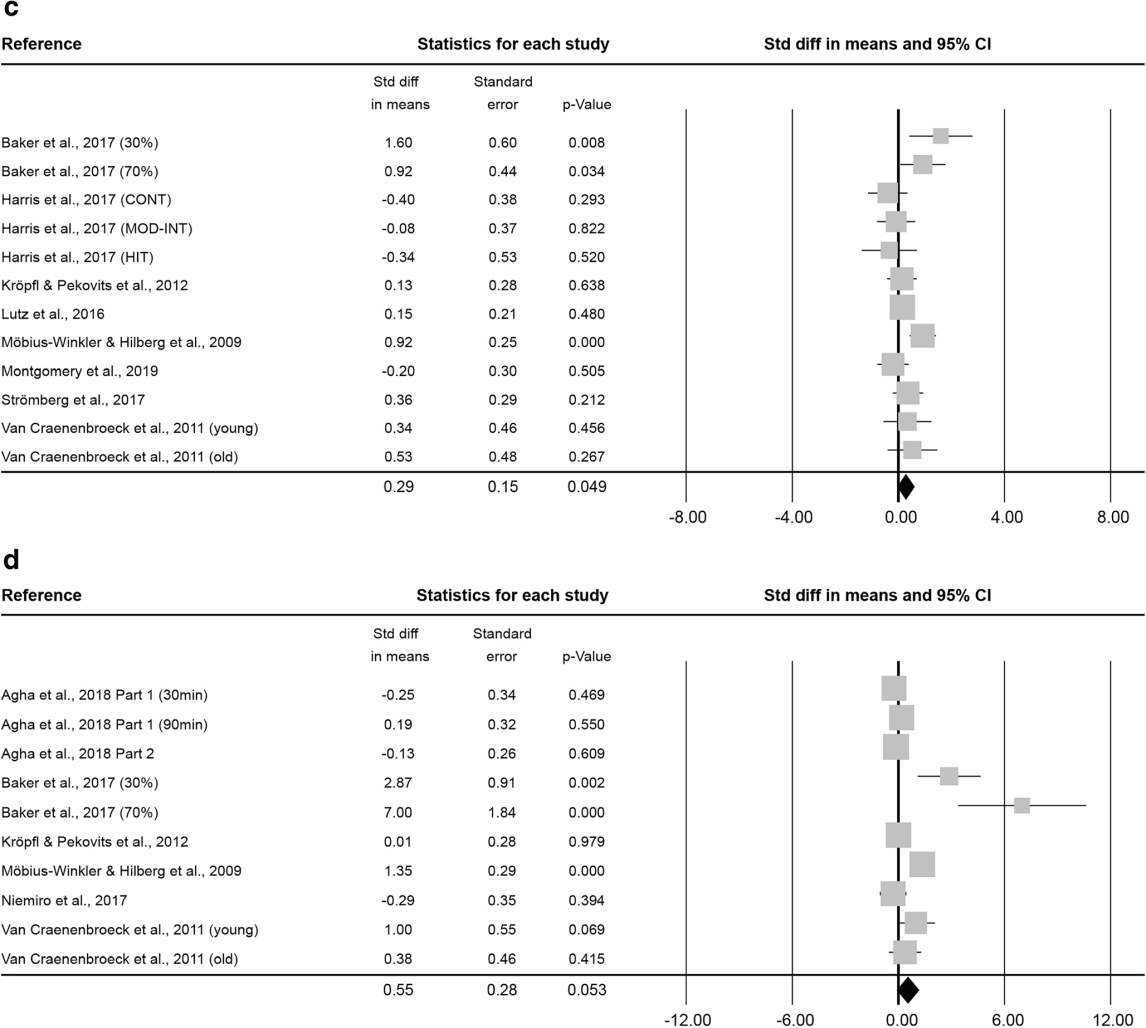

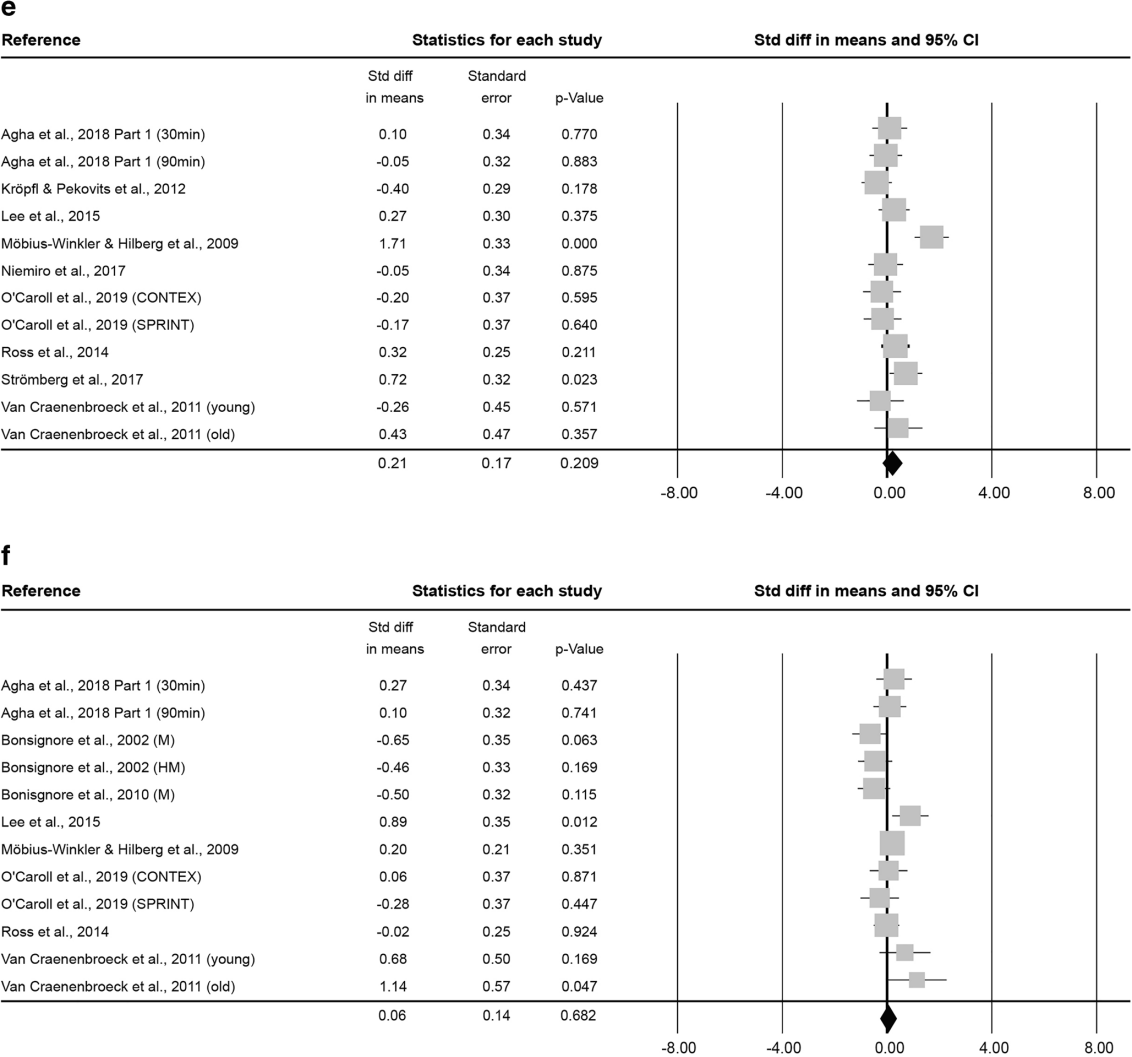
Forest plot of enSCs 0–5 min (**a**), 6–20 min (**b**), 0.5 h (**c**), 1 h (**d**), 2 h (**e**) and 3–96 h post-exercise (**f**). Std diff = standardized difference, CI = confidence interval

In the 0–5 min bin, a total of 27 effect sizes, with 344 subjects (87.7 ± 19.2% male, 31.2 ± 13.9 years old, BMI of 23.9 ± 1.9 kg·m^− 2^) were included in the meta-analysis. The exercise interventions had a mean duration of 2.2 ± 6.6 h at an intensity of 2.7 ± 0.6. The combined Std diff in means (± SE) showed a significant overall increase in enSCs directly after exercise (0.64 ± 0.16, *p* < 0.001; Fig. 2a).

The 6–20 min bin included 29 effect sizes, with an overall number of 284 subjects (91.5 ± 6.4% male, 32.9 ± 15.4 years old, BMI of 23.4 ± 1.0 kg·m^− 2^) and exercise interventions lasting on average 42.7 ± 53.9 min at an intensity of 2.7 ± 0.6. The combined Std diff in means was 0.42 ± 0.11 (*p* < 0.001, Fig. 2b).

At 0.5 h after exercise, 12 effect sizes were assessed. This resulted in 99 subjects (75 ± 45.2% male, 38.3 ± 20.2 years old, BMI of 24.0 ± 1.2 kg·m^− 2^). On average, exercise interventions lasted 55.4 ± 70.3 min at an intensity of 2.3 ± 0.8. Cell numbers increased significantly pre- to 0.5 h post-exercise, as indicated by a combined Std diff in means of 0.29 ± 0.15 (*p* = 0.049, Fig. 2c).

At 1 h after exercise, 10 effect sizes, including 81 subjects (89.0 ± 19.5% male, 30.9 ± 14.7 years old, BMI of 24.0 ± 1.3 kg·m^− 2^) were included. Exercise interventions lasted on average 1.1 ± 1.2 h at a mean intensity of 2.6 ± 0.7. The combined Std diff in means was not significant (0.55 ± 0.28, *p* = 0.053; Fig. 2d).

At 2 h after exercise, 12 effect sizes, including 102 subjects (86.7 ± 20.1% male, 30.4 ± 13.3 years old, BMI of 24.0 ± 1.3 kg·m^− 2^) were analysed. The average intervention lasted 1.1 ± 1.2 h at an intensity of 2.6 ± 0.7. The combined Std diff in means revealed no significant change in cell numbers compared to baseline (0.21 ± 0.17, *p* = 0.209; Fig. 2e).

At 3–96 h after exercise, 12 effect sizes, with a total of 100 subjects (86.7 ± 20.1% male, 34.7 ± 14.3 years old, BMI of 24.0 ± 1.3 kg·m^− 2^) were analysed. Mean exercise time was 1.7 ± 1.5 h at an intensity of 2.7 ± 0.7. The combined Std diff in means was not significant (0.06 ± 0.14, *p* = 0.682; Fig. 2f).

### Endothelial Stem and Progenitor Cells (ESCs)

**Fig. 3:**
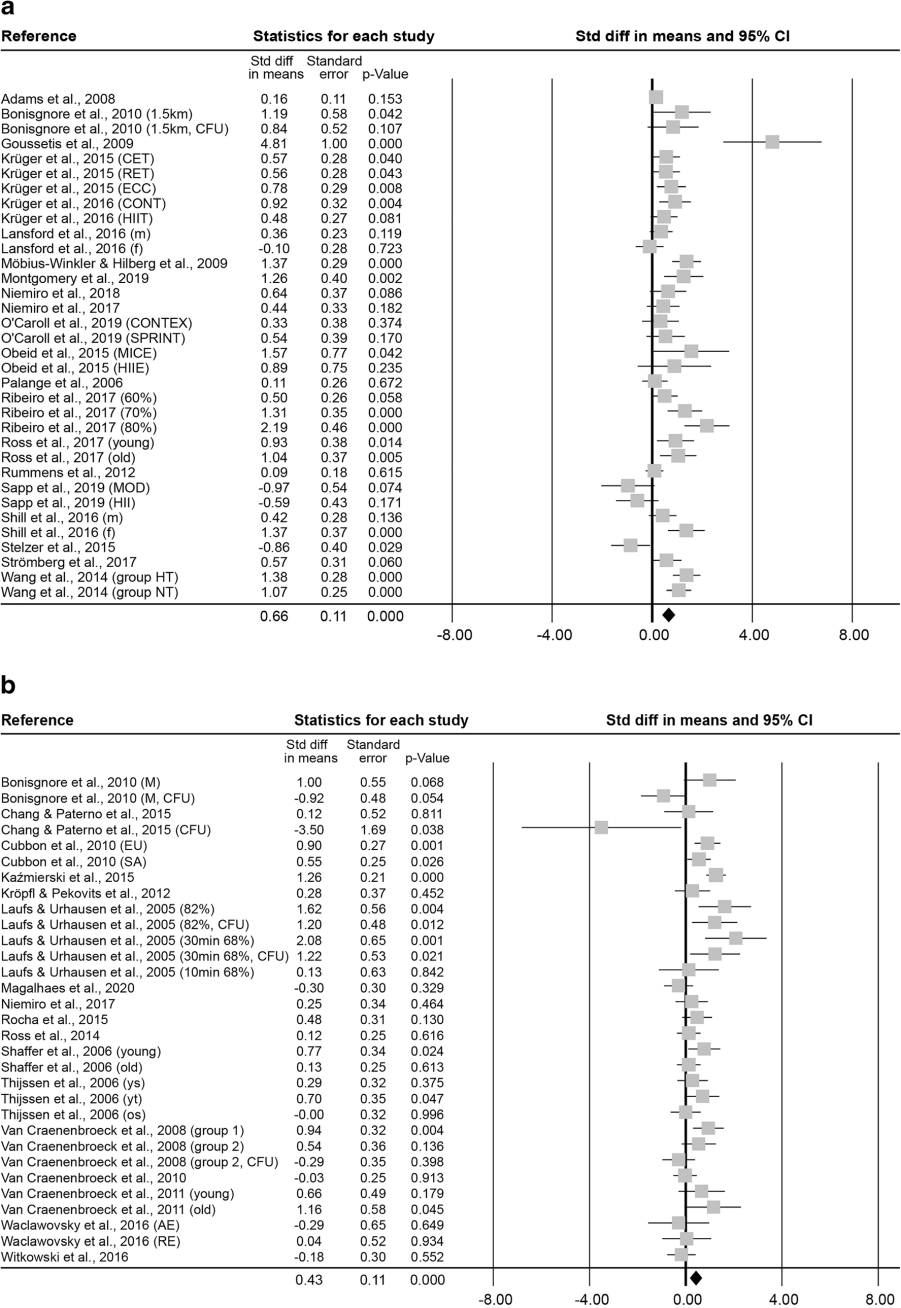

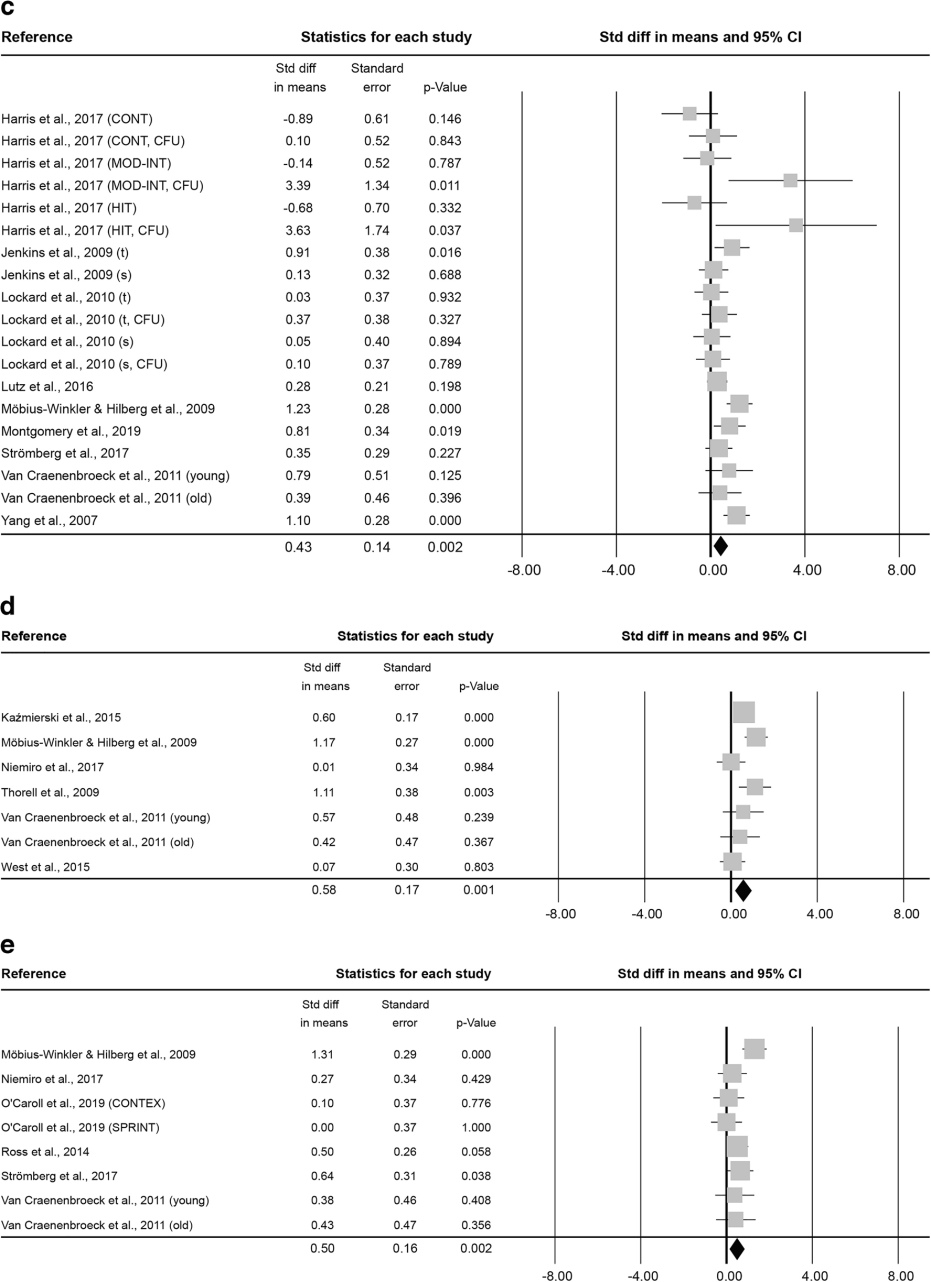

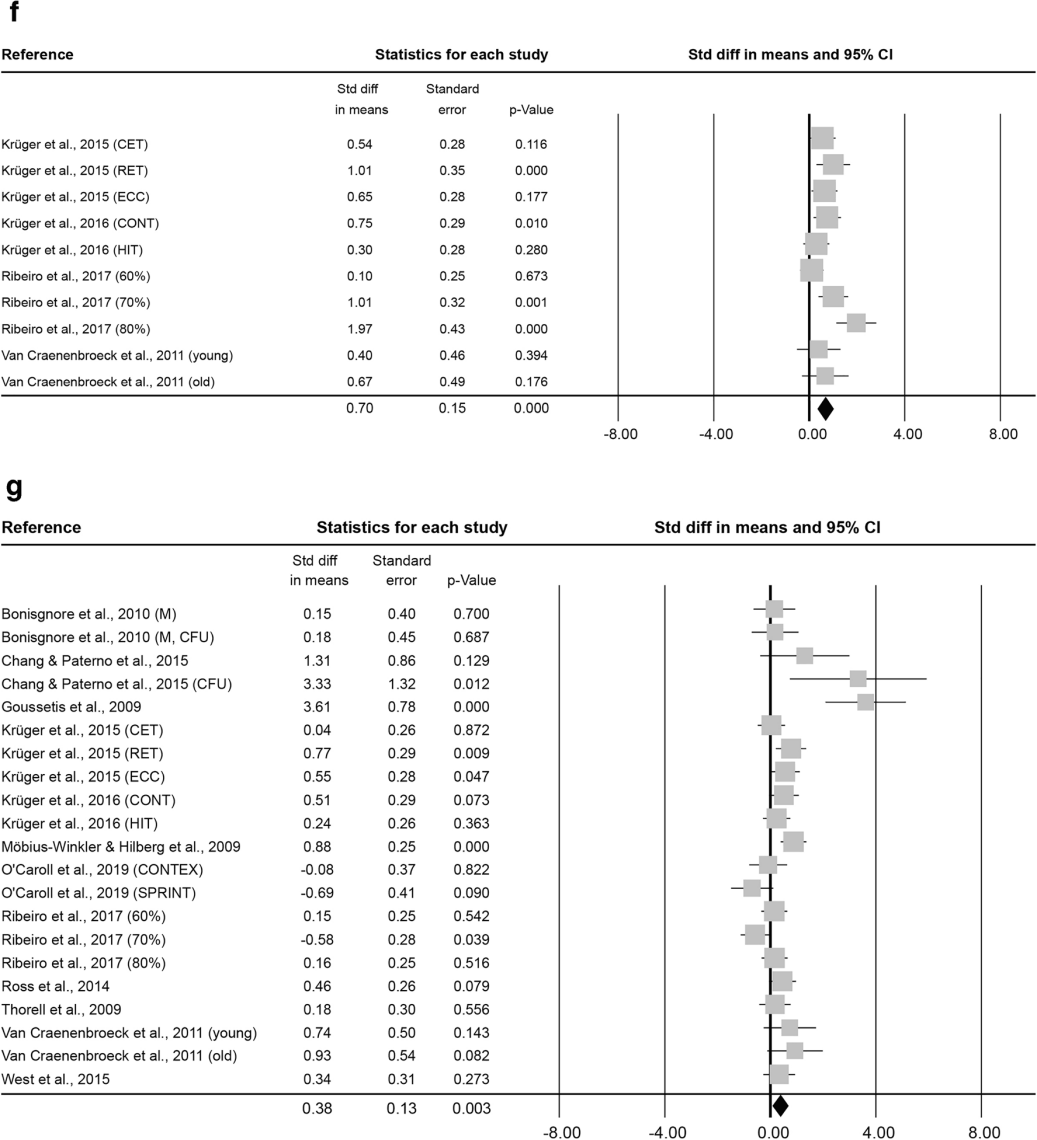
Forest plot of ESCs 0–5 min (**a**), 6–20 min (**b**), 0.5 h (**c**), 1 h (**d**), 2 h (**e**), 3–8 h (**f**) and 12–48 h post-exercise (**g**). Std diff = standardized difference, CI = confidence interval

At 0–5 min after exercise, 34 effect sizes, with an overall number of 410 subjects (73.8 ± 40.0% male, 30.1 ± 13.2 years old, BMI of 23.5 ± 1.8 kg·m^− 2^) were included in the meta-analysis of ESCs. Subjects exercised 2.8 ± 7.9 h on average at an intensity of 2.8 ± 0.5. This resulted in a significant combined Std diff in means of 0.66 ± 0.11 (*p* < 0.001, Fig. 3a).

At 6–20 min after exercise, 31 effect sizes were analysed. The 252 included subjects (93.2 ± 14.1% male, 34.1 ± 14.1 years old, BMI of 23.6 ± 1.4 kg·m^− 2^) exercised on average 43.2 ± 49.4 min at an intensity of 2.8 ± 0.5. The combined Std diff in means showed a significant increase in cell numbers (0.43 ± 0.11, *p* < 0.001, Fig. 3b).

At 0.5 h after exercise, 19 effect sizes, with 133 subjects ( 68.4 ± 47.8% male, 47.6 ± 19.9 years old, BMI of 23.8 ± 1.3 kg·m^− 2^) were included. After a mean exercise time of 43.7 ± 53.2 min at an intensity of 2.6 ± 0.6, the combined Std diff in means showed a significant increase of a point estimate of 0.43 ± 0.14 (*p* = 0.002, Fig. 3c).

At 1 h after exercise, 7 effect sizes, including 84 subjects (86.3 ± 29.1% male, 35.6 ± 17.2 years old, 23.9 ± 2.4 kg·m^− 2^ BMI) were analysed. The interventions lasted 1.6 ± 1.6 h on average at a mean intensity of 2.9 ± 0.4. Combined Std diff in means showed a significant increase in cell numbers 1 h after exercise (0.58 ± 0.17, *p* = 0.001; Fig. 3d).

At 2 h after exercise, 8 effect sizes, with a total of 68 subjects (91.7 ± 15.4% male, 31.9 ± 16.5 years old, BMI of 24.2 ± 1.5 kg·m^− 2^) were included in the meta-analysis of ESCs. Interventions lasted on average 1.2 ± 1.4 h at an intensity of 2.5 ± 0.8 and a significant combined Std diff in means of 0.50 ± 0.16 (*p* = 0.002, Fig. 3e) was found.

At 3–8 h after exercise, 10 effect sizes, including a total of 105 subjects (70.0 ± 48.3% male, 28.4 ± 15.4 years old, BMI of 23.3 ± 1.3 kg·m^− 2^) were analysed. Exercise interventions lasted on average 27.5 ± 2.8 min at an intensity of 2.9 ± 0.3. The analysis resulted in a significant increase indicated by a combined Std diff in means of 0.70 ± 0.15 (*p* < 0.001, Fig. 3f).

At 12–48 h after exercise, 21 effect sizes, with 190 subjects (77.8 ± 38.7% male, 30.7 ± 11.7 years old, BMI of 23.8 ± 1.3 kg·m^− 2^) were included. They involved 2.6 ± 7.2 h of exercise at an intensity of 2.8 ± 0.5 on average. Results showed a significant combined Std diff in means (0.38 ± 0.13, *p* = 0.003, Fig. 3g).

### Hematopoietic Stem and Progenitor Cells (HSCs)

**Fig. 4:**
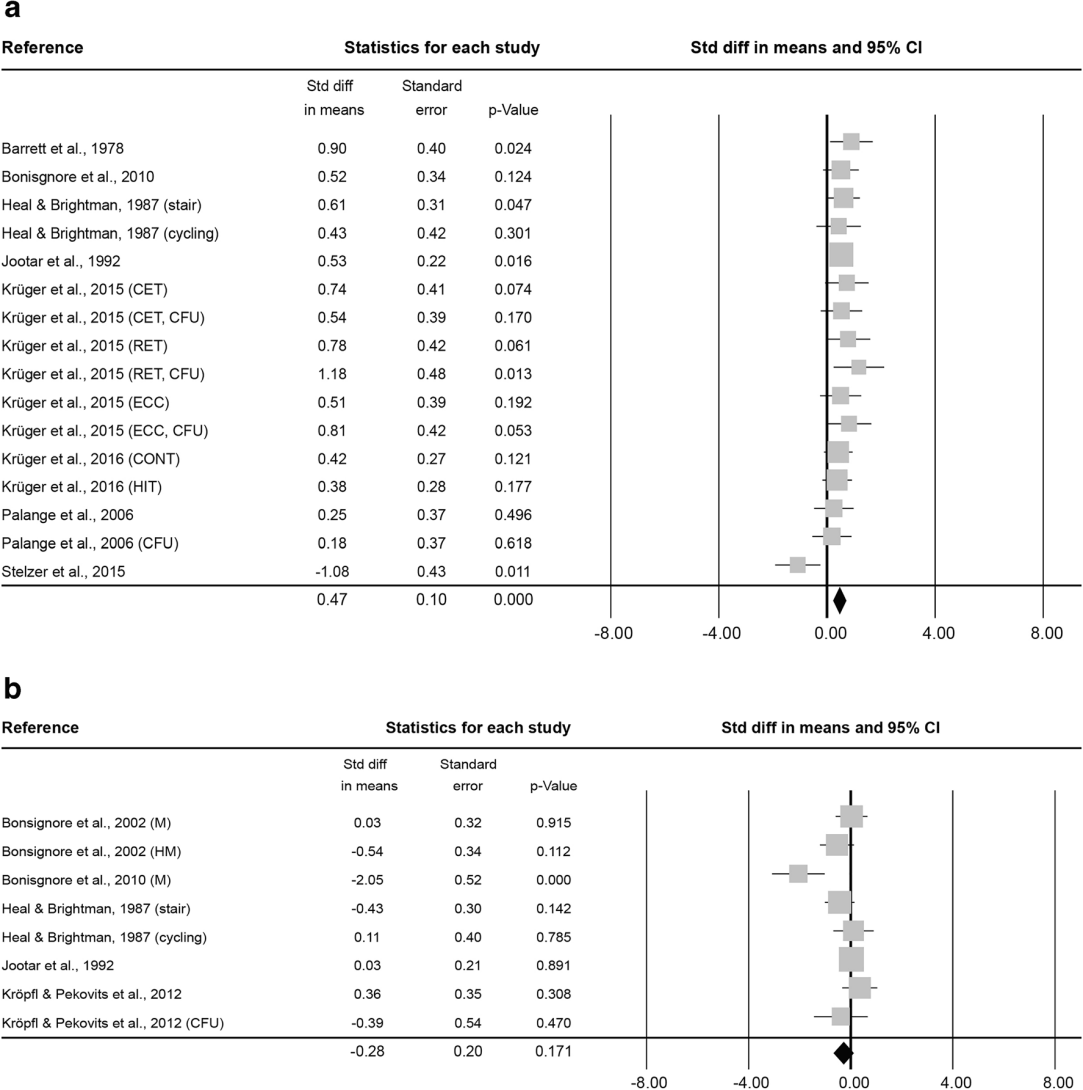

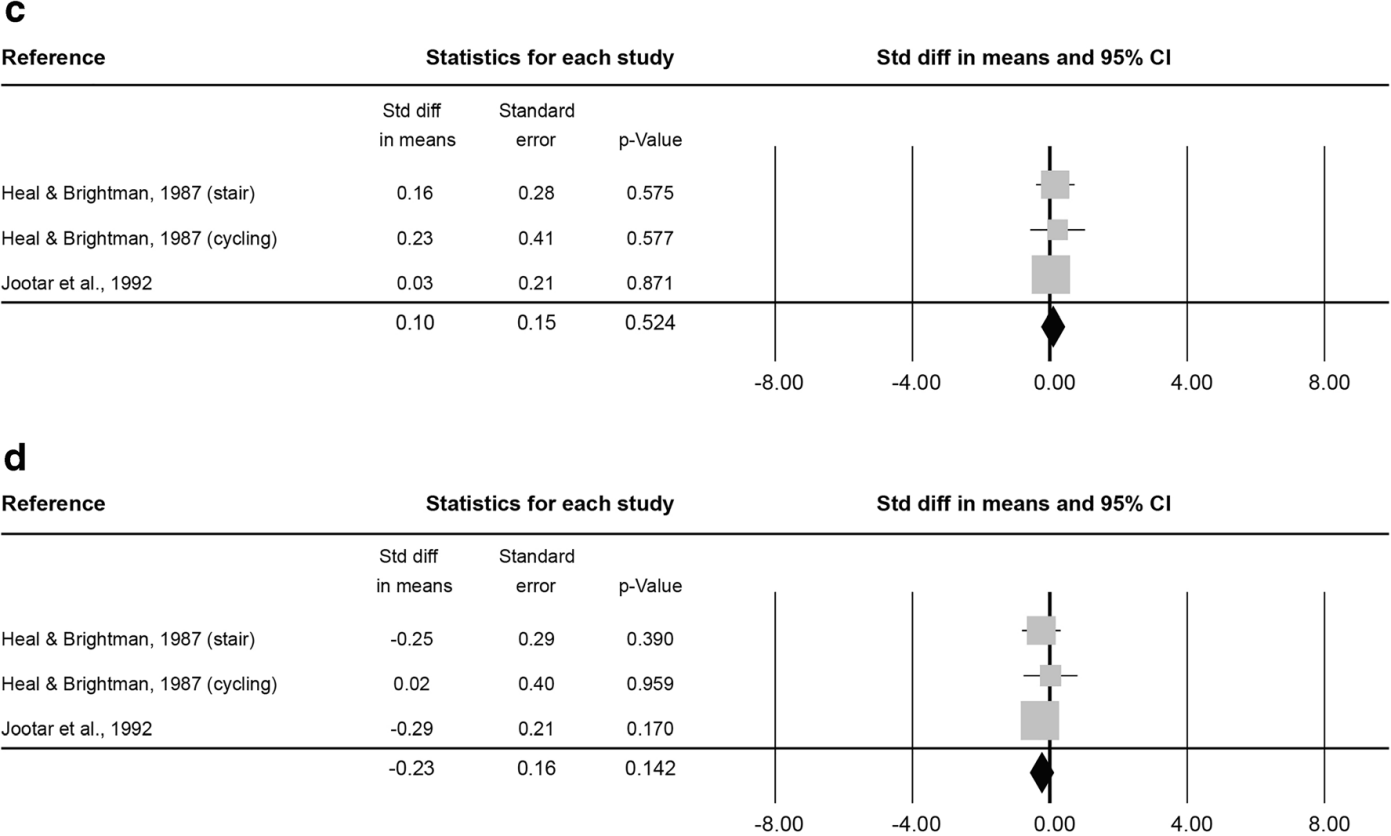

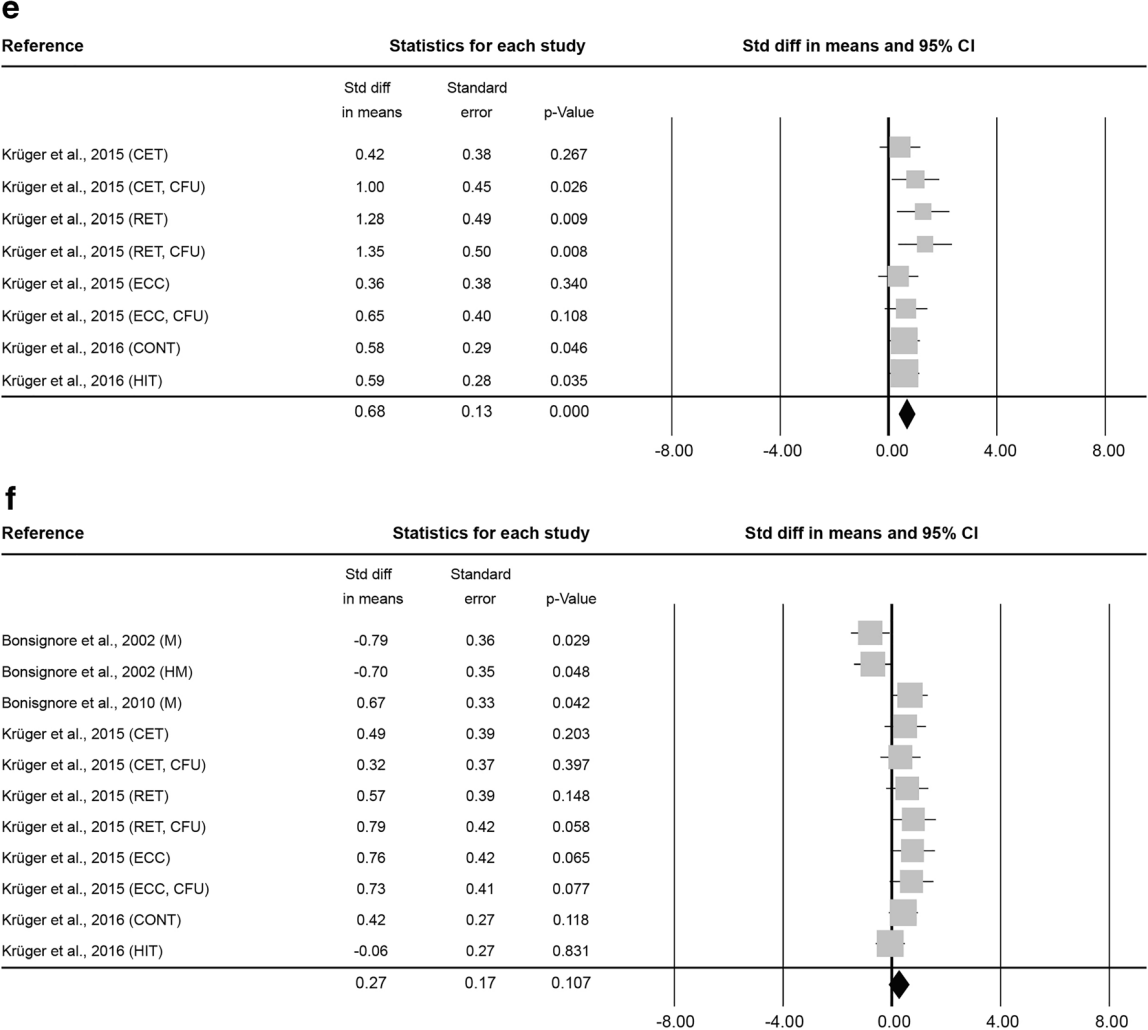
Forest plot of HSCs 0–5 min (**a**), 6–20 min (**b**), 0.5 h (**c**), 1 h (**d**), 3 h (**e**) and 12–24 h post-exercise (**f**). Std diff = standardized difference, CI = confidence interval

At 0–5 min after exercise, 16 analysed effect sizes included a total of 127 subjects (86.2 ± 21.0% male, 33.8 ± 12.6 years old, BMI of 24.4 ± 0.8 kg·m^− 2^). Exercise lasted on average 2.4 ± 7.9 h at an intensity of 2.9 ± 0.3. The combined Std diff in means of 0.47 ± 0.10 represents a significant overall increase in HSC number directly after exercise (*p* < 0.001, Fig. 4a).

At 6–20 min after exercise, 8 effect sizes, resulting in a total of 69 subjects (84.7 ± 23.6% male, 34.2 ± 8.7 years old, BMI 23.2 ± 0.3 kg·m^− 2^) were included. The average intervention lasted 1.2 ± 1.4 h at an intensity of 2.8 ± 0.5. No significant combined Std diff in means was found (-0.28 ± 0.20, *p* = 0.171; Fig. 4b).

At 0.5 h after exercise, 3 effect sizes, including 34 subjects ( 59.3 ± 20.0% male, 31.8 ± 1.2 years old) were analysed. The average exercise intervention lasted 13.5 ± 10.3 min at an intensity of 2.3 ± 0.6. The combined Std diff in means showed no changes in HSC numbers 0.5 h after exercise compared to baseline (0.10 ± 0.15, *p* = 0.524; Fig. 4c). No information was provided on subjects’ BMI or height/weight.

At 1 h after exercise, another 3 effect sizes, including a total of 84 subjects (59.3 ± 20.0% male, 31.8 ± 1.2 years old) were analysed. On average, exercise interventions lasted 13.5 ± 10.3 min at a mean intensity of 2.3 ± 0.6. The analysis of the combined Std diff in means found no significant effect of exercise on circulating HSC numbers measured 1 h after exercise (-0.23 ± 0.16, *p* = 0.142; Fig. 4d). No information was provided on subjects’ BMI or height/weight.

At 3 h after exercise, 8 effect sizes included a total of 59 subjects (100.0 ± 0.0% male, 25.9 ± 0.4 years old, BMI of 24.2 ± 0.2 kg·m^− 2^). The average intervention lasted 39.2 ± 22.8 min and was conducted at a vigorous intensity of 3.0 ± 0.0. The analysis found a significant combined Std diff in means 3 h post-exercise (0.68 ± 0.13, *p* < 0.001; Fig. 4e).

At 12–24 h after exercise, 11 effect sizes, with 84 subjects (100.0 ± 0.0% male, 30.4 ± 8.6 years old, BMI of 24.0 ± 0.4 kg·m^− 2^ ) were included. After an average intervention of 1.2 ± 1.1 h exercising at an intensity of 3.0 ± 0.0, the combined Std diff in means showed no significant change in HSC numbers (0.27 ± 0.17, *p* = 0.107; Fig. 4f).

### Mesenchymal Stem and Progenitor Cells (MSCs)

**Fig. 5:**
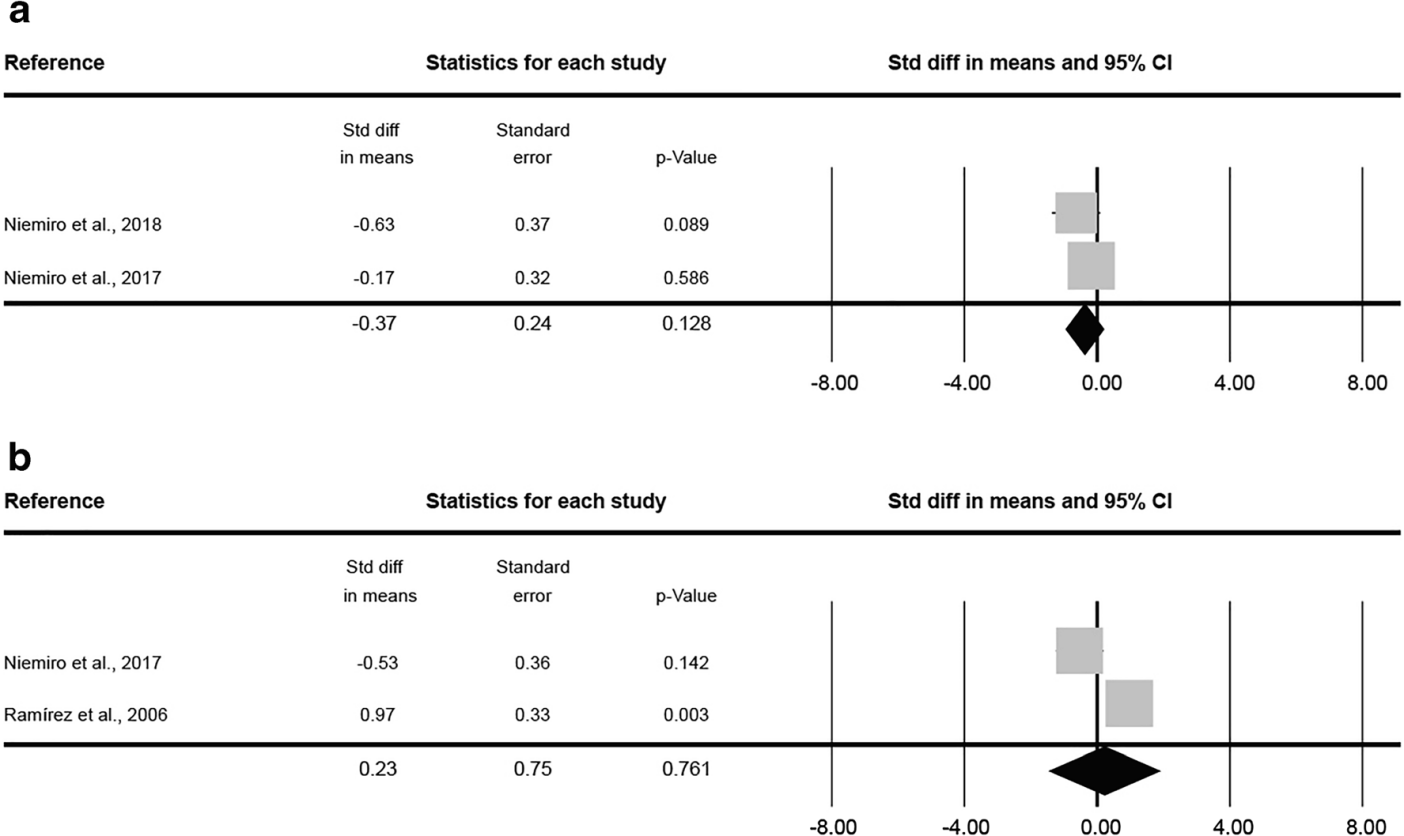
Forest plot of MSCs 0–5 min (**a**) and 2 h post-exercise (**b**). Std diff = standardized difference, CI = confidence interval

At 0–5 min after exercise, 2 effect sizes were reported, resulting in a total of 15 subjects (81.3 ± 26.5% male, 26.4 ± 1.6 years old, BMI of 21.9 ± 2.3 kg·m^− 2^). Interventions lasted on average 56.0 ± 5.7 min and were conducted at a vigorous intensity of 3.0 ± 0.0. The combined Std diff in means indicated no significant change in cell numbers (-0.37 ± 0.24, *p* = 0.128; Fig. 5a).

At 2 h after exercise, 2 effect sizes, with 18 subjects (100.0 ± 0.0% male, 30.2 ± 6.9 years old, BMI of 23.5 ± 0.0 kg·m^− 2^). Interventions had a mean duration of 1.0 ± 0.0 h and were conducted at an intensity of 3.0 ± 0.0. The analysis of the combined Std diff in means showed no effect of exercise on MSC numbers 2 h post-intervention (0.23 ± 0.75, *p* = 0.761; Fig. 5b).

### Synthesis of Results

Table 5 shows an overview of all meta-analyses including the respective combined effects and measures of heterogeneity.

**Table 5:** Summary of combined effects and heterogeneity

Outcome		N	Combined effects			Heterogeneity			
Sub-group	Time bin		Std diff in means	95% CI	*p*-value	*Q*-value	*df* (*Q*)	*p*-value	*I*^*2*^ in %
enSCs	0 - 5 min	27	0.64	0.32 - 0.97	**0.000**	197.0	26	0.000	86.8
	6 - 20 min	29	0.42	0.19 - 0.64	**0.000**	95.1	28	0.000	70.6
	0.5 h	12	0.29	0.00 - 0.57	**0.049**	22.6	11	0.020	51.3
	1 h	10	0.55	-0.01 - 1.10	0.053	45.4	9	0.000	80.2
	2 h	12	0.21	-0.12 - 0.54	0.209	32.5	11	0.001	66.1
	3 - 96 h	12	0.06	-0.21 - 0.33	0.682	22.0	11	0.024	50.1
ESCs	0 - 5 min	34	0.66	0.45 - 0.87	**0.000**	128.6	33	0.000	74.3
	6 - 20 min	31	0.43	0.21 - 0.64	**0.000**	80.9	30	0.000	62.9
	0.5 h	19	0.43	0.16 - 0.70	**0.002**	38.4	18	0.003	53.1
	1 h	7	0.58	0.24 - 0.92	**0.001**	12.5	6	0.052	52.0
	2 h	8	0.50	0.19 - 0.81	**0.002**	11.6	7	0.113	39.8
	3 - 8 h	10	0.70	0.40 - 0.99	**0.000**	19.2	9	0.023	53.2
	12 - 48 h	21	0.38	0.13 - 0.62	**0.003**	55.7	20	0.000	64.1
HSCs	0 - 5 min	16	0.47	0.28 - 0.67	**0.000**	19.8	15	0.181	24.1
	6 - 20 min	8	-0.28	-0.67 - 0.12	0.171	19.1	7	0.008	63.3
	0.5 h	3	0.10	-0.20 - 0.40	0.524	0.2	2	0.884	0.0
	1 h	3	-0.23	-0.53 - 0.08	0.142	0.5	2	0.790	0.0
	3 h	8	0.68	0.42 - 0.94	**0.000**	5.1	7	0.644	0.0
	12 - 24 h	11	0.27	-0.06 - 0.60	0.107	24.5	10	0.006	59.1
MSCs	0 - 5 min	2	-0.37	-0.84 - 0.11	0.128	0.9	1	0.351	0.0
	2 h	2	0.23	-1.24 - 1.70	0.761	9.5	1	0.002	89.5

Std diff = standardized difference, CI = confidence interval df = degrees of freedom, N = number of effect sizes included. Significant *p* - values for effect sizes are in bold

**Fig. 6:**
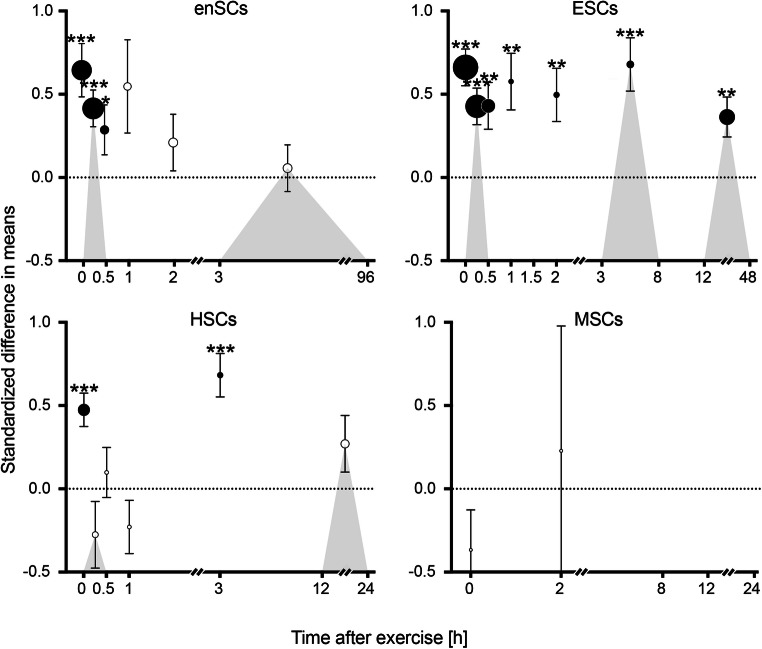
Graphical summary of the main outcomes. enSCs = early and non-specified stem and progenitor cells, ESCs = endothelial stem and progenitor cells, HSCs = hematopoietic stem and progenitor cells, MSCs = mesenchymal stem and progenitor cells. Data is given as Std diff in means ± SE, with the size of each datapoint representing the number of included effect sizes in the respective meta-analysis. Significant effects are depicted by filled symbols and level of significance: *p < 0.050, **p < 0.010, ***p < 0.001

An overview of the average Std diff in means (change pre-to-post exercise) of stem and progenitor cell numbers at different times after exercise is provided in Fig. 6. Each datapoint represents the effect of exercise found for the respective cell population at the specified timepoint/-range and thus shows the resulting combined effect size of one individual meta-analysis.

Assuming baseline values of 0.1–6.0% mononuclear blood cells (MNCs) with a maximal SD of ± 0.2% for EPCs and 0.010–0.2% MNCs ± max 0.01% for HSCs [85], and using formulas 2–4, the obtained Std diff in means of 0.66 for ESCs and 0.47 for HSCs, reflecting the true effects of exercise on stem cell mobilization directly after exercise, represent an increase of 0.15% and 0.005% MNCs, respectively. Assuming an estimate of approximately 2 × 10^6^ MNCs/ml blood [86], this equals an absolute increase of 3000 cells/ml for ESCs and 100 cells/ml for HSCs.

### Risk of Bias across Studies

The following five outcomes included 20 or more effect sizes and were thus analyzed for publication bias:

### Early and non-specified Stem and Progenitor Cells (enSCs), 0–5 min post

Even though the funnel plot did not look noticeably asymmetric, Egger’s regression test resulted in a significant *p* - value of 0.000. This was, however, not confirmed by the rank correlation test (Kendall’s tau = 0.09, *p* = 0.252). Orwin’s *Fail - safe N* showed that 167 additional studies with an effect size of 0.0 would need to be added in order to render the cumulative effect size trivial (≤ 0.05). The trim and fill method suggested that 5 studies reporting negative changes in cell numbers were missing, and that their inclusion might change the effect size (95% CI) of 0.64 (0.32–0.97) to a computed effect of 0.53 (0.22–0.84).

### Early and non-specified Stem and Progenitor Cells (enSCs), 6–20 min post

Egger’s test resulted in a significant *p* - value (*p* = 0.031), while the rank correlation test failed to show a significant correlation between the inversed SE and the effect size (Kendall’s tau = 0.19, *p* = 0.080). An additional 160 studies were predicted by Orwin’s *Fail - safe N* to be necessary to render the combined effect size trivial (≤ 0.05). The inclusion of 3 missing studies to the left of the mean were suggested by the trim and fill test which would change the effect size from 0.42 (0.19–0.64) to 0.30 (0.07–0.54).

### Endothelial Stem and Progenitor Cells (ESCs), 0–5 min post

Asymmetry of the funnel plot was confirmed by both, Egger’s test (*p* = 0.004) and the rank correlation test (Kendall’s tau = 0.23, *p* = 0.027). Orwin’s *Fail - safe N* suggested that the inclusion of an additional 313 studies with an effect size of 0.0 point estimates is needed to render the overall effect size insignificant (≤ 0.05). The trim and fill method calculated a number of 8 studies to be missing and computed a new effect size of 0.41 (0.19–0.64) (compared to 0.66 (0.45–0.87)) if they were to be included.

### Endothelial Stem and Progenitor Cells (ESCs), 6–20 min post

The funnel plot showed a very apparent symmetry, which was supported by both, Egger’s regression test (*p* = 0.403) and the rank correlation test (Kendall’s tau = 0.09, *p* = 0.238). Further, Orwin’s *Fail - safe N* yielded 234 studies would need to be added to render the effect size meaningless (≤ 0.05) and the trim and fill method found 0 studies to be missing, leaving the effect size of 0.43 (0.21–0.64) unchanged.

### Endothelial Stem and Progenitor Cells (ESCs), 12–48 h post

The regression test resulted in a significant *p* - value of 0.022, indicating asymmetry in the funnel plot. This was however not confirmed by the rank correlation test (Kendall’s tau = 0.25, *p* = 0.055),. 113 studies were found by Orwin’s *Fail - safe N* and the trim and fill method suggested 3 additional studies to be included to the left of the mean, which would change the existing, significant effect size of 0.38 (0.13–0.62) to a non-significant effect of 0.26 (-0.01–0.55).

### Sensitivity analyses

The following five outcomes included 20 or more effect sizes and were thus subjected to sensitivity analyses:

### Early and non-specified Stem and Progenitor Cells (enSCs), 0–5 min post

The one-effect-size-removed analysis showed no change in significance of the combined Std diff in means (*p* < 0.001 for all 27 effect sizes). Imputing a correlation coefficient of r = 0.5 or r = 0.7 instead of r = 0.6, did not change the significance of the obtained effect size either (0.5: 0.67 ± 0.11, *p* < 0.001; 0.7: 0.63 ± 0.10, *p* < 0.001).

### Early and non-specified Stem and Progenitor Cells (enSCs), 6–20 min post

The removal of any of the 29 included effect sizes in the one-effect-size-removed analysis did not change the significance of the combined Std diff in means. Similarly, the substitution of r = 0.6 with r = 0.5 or r = 0.7 did not change the significance of the outcome (0.5: 0.42 ± 0.12, *p* < 0.001; 0.7: 0.40 ± 0.11, *p* < 0.001).

### Endothelial Stem and Progenitor Cells (ESCs), 0–5 min post

The one-effect-size-removed analysis showed no change in significance of the combined Std diff in means (*p* < 0.001 for all 34 effect sizes)., neither did any applied adjustments to the correlation coefficient (0.5: 0.68 ± 0.11, *p* < 0.001; 0.7: 0.63 ± 0.10, *p* < 0.001).

### Endothelial Stem and Progenitor Cells (ESCs), 6–20 min post

The originally obtained *p*-value of 0.000 did not change upon the application of the one-effect-size-removed analysis, no matter which one of the 31 included effect sizes was removed. Also adjusting r from 0.6 to 0.5 or 0.7 did not result in any changes of the level of significance (0.5: 0.44 ± 0.11, *p* < 0.001; 0.7: 0.41 ± 0.11, *p* < 0.001).

### Endothelial Stem and Progenitor Cells (ESCs), 12–48 h post

The one-effect-size-removed analysis did not change the significance of the combined Std diff in means for any of the 21 effect sizes. Level of significance was also not substantially altered when imputing correlation coefficients of 0.5 or 0.7 (0.5: 0.37 ± 0.13, *p* = 0.004; 0.7: 0.37 ± 0.12, *p* = 0.002).

### Moderator variables

All of the five outcomes including a minimum of 20 effect sizes also showed a significant heterogeneity of *I*^*2*^ > 50% in their results. They thus all qualified for further analysis of moderator variables via subgroup analysis and meta-regression. A detailed overview of the results is given in Table 6.

**Table 6:** Moderator variable analyses

Outcome	Results continuous variables				Results categorical variables					
	Moderator variable	n	% variance explained	*p*-value	Moderator variable	Subgroup	n	Std diff in means *±* SE	*p*-value effect size (within the subgroup)	*p*-value heterogeneity (across the subgroups)
SCs, 0–5 min post	Sex of subjects (%male)	25	0	0.700	Intervention intensity	Low	2	0.52 ± 0.64	0.419	0.947
						Moderate	4	0.55 ± 0.44	0.206	
	Age of subjects	27	25	0.118		Vigorous	21	0.68 ± 0.19	0.000	
					Modality of intervention	Cycling	19	0.76 ± 0.18	0.000	0.485
	BMI of subjects	26	0	0.568		Running	5	0.43 ± 0.34	0.202	
						Resistance	2	-0.01 ± 0.52	0.984	
	Intervention duration	23	7	0.199		Other	1	0.68 ± 0.75	0.369	
					Baseline physical activity level of subjects	Sedentary	5	1.02 ± 0.36	0.004	0.244
	Intervention load	23	8	0.181		Active	13	0.73 ± 0.24	0.002	
SCs, 6–20 min post	Sex of subjects (%male)	27	0	0.957	Intervention intensity	Low	2	-0.09 ± 0.45	0.845	0.451
						Moderate	4	0.31 ± 0.33	0.342	
	Age of subjects	29	5	0.210		Vigorous	23	0.48 ± 0.13	0.000	
					Modality of intervention	Cycling	15	0.41 ± 0.16	0.010	0.229
	BMI of subjects	26	0	0.224		Running	10	0.33 ± 0.19	0.090	
						Resistance	2	0.15 ± 0.45	0.732	
	Intervention duration	22	0	0.112		Other	2	1.39 ± 0.50	0.006	
					Baseline physical activity level of subjects	Sedentary	7	0.53 ± 0.25	0.036	0.469
	Intervention load	22	0	0.168		Active	19	0.45 ± 0.14	0.001	
ESCs, 0–5 min post	Sex of subjects (%male)	31	0	0.390	Intervention intensity	Low	1	1.26 ± 0.66	0.054	0.432
						Moderate	6	0.42 ± 0.27	0.122	
	Age of subjects	34	12	0.238		Vigorous	27	0.69 ± 0.12	0.000	
					Modality of intervention	Cycling	20	0.48 ± 0.15	0.001	0.186
	BMI of subjects	33	9	0.088		Running	8	0.89 ± 0.24	0.000	
						Resistance	5	1.09 ± 0.29	0.000	
	Intervention duration	31	1	0.887		Other	1	0.44 ± 0.63	0.485	
					Baseline physical activity level of subjects	Sedentary	10	0.88 ± 0.18	0.000	0.087
	Intervention load	31	2	0.845		Active	17	0.68 ± 0.16	0.000	
ESCs, 6–20 min post	Sex of subjects (%male)	29	0	0.886	Intervention intensity	Low	1	0.13 ± 0.55	0.820	0.853
						Moderate	5	0.44 ± 0.28	0.110	
	Age of subjects	31	0	0.616		Vigorous	25	0.44 ± 0.13	0.001	
					Modality of intervention	Cycling	15	0.37 ± 0.15,	0.016	0.497
	BMI of subjects	29	0	0.881		Running	14	0.57 ± 0.18	0.001	
						Resistance	2	0.10 ± 0.42	0.823	
	Intervention duration	22	0	0.430		Other	0			
					Baseline physical activity level of subjects	Sedentary	8	0.37 ± 0.22	0.099	0.909
	Intervention load	22	0	0.434		Active	18	0.43 ± 0.15	0.003	
ESCs, 12–48 h post	Sex of subjects (%male)	20	46	**0.010**	Intervention intensity	Low	1	0.46 ± 0.54	0.398	0.920
						Moderate	2	0.52 ± 0.38	0.174	
	Age of subjects	21	8	0.110		Vigorous	18	0.36 ± 0.15	0.014	
					Modality of intervention	Cycling	9	0.29 ± 0.19	0.125	0.206
	BMI of subjects	20	1	0.421		Running	7	0.77 ± 0.26	0.003	
						Resistance	5	0.19 ± 0.24	0.418	
	Intervention duration	19	59	**0.000**		Other	0			
					Baseline physical activity level of subjects	Sedentary	9	0.25 ± 0.18	0.172	0.497
	Intervention load	19	54	**0.000**		Active	7	0.59 ± 0.22	0.008	

Std diff = standardized difference, SE = standard error, N = number of effect sizes included in the analysis. Significant *p* - values of moderating effects of the variables on the effect sizes are in bold

### Early and non-specified Stem and Progenitor Cells (enSCs), 0–5 min post

Meta-regressions revealed that none of the investigated moderator variables could significantly predict changes in enSCs measured between 0 and 5 min post-exercise compared to baseline (Sex: *p* = 0.700, Age: *p* = 0.118, BMI: *p* = 0.568, Duration of the intervention: *p* = 0.199, Total load of the intervention: *p* = 0.181). Correcting for the variables removed 0% of the between-study variance, except for “age”, which was responsible for 25% of the variance.

Subgroup analyses categorizing effect sizes based on the intensity of the intervention, its modality or the baseline physical activity level of the subjects revealed that none of the categories within a subgroup differed significantly in their outcomes (Intensity: *p* = 0.947, Modality: *p* = 0.485, Baseline physical activity level: *p* = 0.244).

### Early and non-specified Stem and Progenitor Cells (enSCs), 6–20 min post

Meta-regressions revealed that none of the investigated moderator variables could significantly predict changes in enSCs measured between 6 and 20 min post-exercise compared to baseline (Sex: *p* = 0.957, Age: *p* = 0.210, BMI: *p* = 0.224, Duration of the intervention: *p* = 0.112, Total load of the intervention: *p* = 0.168). Correcting for the variables removed 0% of the between-study variance, except for “age”, which was responsible for 5% of the variance.

Subgroup analyses categorizing effect sizes based on the intensity of the intervention, its modality or the baseline physical activity level of the subjects revealed that none of the categories within a subgroup differed significantly in their outcomes (Intensity: *p* = 0.451, Modality: *p* = 0.229, Baseline physical activity level: *p* = 0.469).

### Endothelial Stem and Progenitor Cells (ESCs), 0–5 min post

Neither of the subject- or intervention-defining parameters significantly correlated with the changes in ESCs directly after exercise, as computed via meta-regressions (Sex: *p* = 0.390, Age: *p* = 0.238, BMI: *p* = 0.088, Duration: *p* = 0.887, Load: *p* = 0.845).

Also, when divided into subgroups, none of the assessed parameters seemed to have a significant influence on the effect size (Intensity: *p* = 0.432, Modality: *p* = 0.186, Baseline physical activity level: *p* = 0.087).

### Endothelial Stem and Progenitor Cells (ESCs), 6–20 min post

Similarly as for ESCs measured directly after exercise, changes in ESC numbers assessed between 6 and 20 min post were not significantly influenced by the percentage of males in the study cohort (*p* = 0.886), subjects’ mean age (*p* = 0.616) or their average BMI (*p* = 0.881). As for the intervention-defining parameters, neither the duration (*p* = 0.430), nor the load (*p* = 0.434) turned out to be significant predictor variables either.

Subgroup analysis showed a comparable picture: effect sizes for the three different intensity-groups (*p* = 0.853), as well as for the groups representing the different exercise modalities (*p* = 0.497), or for groups formed based on subjects’ baseline physical activity level (*p* = 0.909) did not differ significantly.

### Endothelial Stem and Progenitor Cells (ESCs), 12–48 h post

While age (*p* = 0.110) and BMI (*p* = 0.421) of the subject cohort were shown to not significantly correlate to the effect size, %male subjects (*p* = 0.010, 46% variance explained), intervention duration (*p* < 0.001, 59% variance explained) and load (*p* < 0.001, 54% variance explained) turned out to be significant predictor variables for the change in ESC numbers measured 12–48 h after exercise. They all positively correlated with the effect size.

The categorical variables however, did not influence the outcome to a significant extent (Intensity: *p* = 0.920, Modality: *p* = 0.206, Baseline physical activity level: *p* = 0.497).

## Discussion

### Main Outcomes

Most of the overall exercise-induced changes in stem and progenitor cell numbers had a Std diff in means of around 0.20–0.70, representing a small to medium effect size [87]. We computed the obtained Std diff in means of 0.66 for ESCs and 0.47 for HSCs, reflecting the true effects of exercise on stem cell mobilization directly after exercise to represent an absolute increase of 3000 cells/ml for ESCs and 100 cells/ml for HSCs. These numbers show that exercise alone cannot nearly serve as a replacement for granulocyte colony stimulating factor (G-CSF), the most commonly used agent in clinical stem cell mobilization [88], yields increases of around 1.2 × 10^5^ CD34^+^-cells/ml following 4 daily injections of 7.5–10.0 µg/kg bodyweight [89], i.e. approximately 100–1000 fold the increase produced by exercise. However, since G-CSF-induced stem cell mobilization fails to evoke sufficient responses in 5–30% of people [90, 91] and is also associated with side-effects [92], physical exercise may be considered as a potential adjuvant in the process of clinical stem cell mobilization [18], potentially enhancing the effect and/or reducing side-effects.

### Early and non-specified Stem and Progenitor Cells (enSCs)

The biggest increase in enSC numbers was found immediately after exercise, with the effect being reduced thereafter, and no longer significant from 1 h post-exercise on. It is disputable, however, whether the lack of significance for the increase 1 h post-exercise with a rather considerable effect size of 0.55 was not solely due to the limited number of included outcomes (n = 10). In any case, SC numbers returned to baseline at least after 2 h of recovery. This recession of cell numbers might be explained by the homing of early stem cells into tissues throughout the body where they mediate repair responses or renew old cells [93].

### Endothelial Stem and Progenitor Cells (ESCs)

Numbers of circulating ESCs increased directly after exercise cessation and remained elevated until 12 h post-exercise and beyond. Possibly, upon initial mobilization, ESCs remain in the blood stream for longer than HSCs or MSCs because their homing destination lies within the peripheral blood stream itself. The positive correlation between duration and load of exercise and the duration of ESC number elevation may suggest that recruitment of ESCs into the blood stream depends on the degree of strain that is applied to the vasculature.

### Hematopoietic Stem and Progenitor Cells (HSCs)

The number of HSCs increased immediately after exercise but was back to baseline already 1 min later, a finding also reported in a previous review [18]. Although HSCs replenish the cells of the blood and therefore would not theoretically need to leave the blood stream, studies have found HSCs to home to distant tissues such as the heart [94], skeletal muscles [95] or the spleen [96]. They primarily migrate to sites of inflammation and damaged tissues to assist in their repair process [97] which could explain the fast drop in HSC numbers.

Interestingly, our results showed a second increase in HSC numbers 3 h post-exercise. This is in line with a finding by Mooren & Krüger, 2015 [98] showing that apoptotic lymphocytes, intravenously injected into a mouse vein, lead to a dose-dependent Sca-1^+^/c-kit^+^ progenitor cell mobilization 3 h after injection. As acute exercise has already been shown to induce lymphocyte apoptosis in circulation and tissue [99, 100], these authors hypothesized that (exercise-induced) apoptotic lymphocytes exert signaling functions relevant to HSCs. However, whether this link is directly applicable to the human system as well, possibly explaining this second increase in HSC number, remains to be evaluated separately.

### Mesenchymal Stem and Progenitor Cells (MSCs)

Unfortunately, only three publications reported numbers of MSCs at 2 timepoints after acute exercise. This does not allow to draw meaningful conclusions regarding kinetics and extent of mobilization. However, results of these single studies look promising and the implication of MSCs not reacting to an acute bout of exercise with an increase in circulating numbers seen for ESCs and HSCs, warrants further investigation. Despite very low concentrations in the peripheral blood (0.001–0.100% of circulating MNCs [101]), MSCs represent an important and promising cell population, with a progeny able to differentiate into cells of the mesenchymal lineage, such as bone, cartilage, tendon, fat, and bone marrow stroma [101]. In the clinical context, these cells could support hematopoiesis and graft facilitation during cell transplantations or the treatment of various immune-related and degenerative diseases [102–104]. Furthermore, it has previously been shown in mice that repeated endurance exercise not only positively affects MSC quantity but also their quality in terms of differentiation potential [105], putting an even larger focus on exercise as a non-pharmacological tool for MSC mobilization. Establishing the true effect of exercise on MSC mobilization thus is of significant importance and therefore, future studies investigating exercise-dependent stem cell mobilization should also include measures of MSCs.

### Risk of Bias across Studies

In order to assess the validity and applicability of the results, the risk of bias among the included studies was tested. The risk of bias within the conducted analysis is subsequently discussed for all qualified outcomes.

Based on visual inspection of the funnel plots and interpretation of the corresponding statistics, the impact of bias across studies was deemed trivial for data included in the meta-analysis of enSCs 0–5 min post-exercise. Studies reporting enSC numbers 6–20 min post-exercise were estimated to show a low degree of bias which, however, is considered to be insignificant to the presented outcome. In the case of ESC reporting, studies included in the meta-analysis 6–20 min post-exercise showed no indication of publication bias, while risk of bias across studies included in the meta-analyses 0–5 min and 12–48 h post-exercise could not be excluded. However, in both cases, judging from the outcome of the Orwin’s *Fail - safe N* and the trim and fill method, the validity of our findings should not be called into question.

### Sensitivity Analyses

To assure a combined result of multiple effect would not represent a single outcome of large magnitude affecting the overall effect in a disproportionate fashion, sensitivity analyses were conducted. For this, the combined effect size was calculated repeatedly, while sequentially excluding single effect sizes one by one.

Furthermore, data was tested for their robustness regarding the influence of the correlation coefficients which was imputed due to primary studies lacking the respective information.

In the present analysis, none of the computed outcomes that were subjected to sensitivity analyses showed significant changes by either test strategy which indicates high robustness of the obtained outcomes against disproportionate influences of individual studies.

### Moderator Variables

In the present analysis we could not confirm outcomes of single studies reporting differences in the mobilization of stem cells depending on subject-related variables such as age [10, 67, 73] or training status [40, 46, 53, 60] nor on intervention-dependent variables such as intensity [39, 41, 60, 61, 64, 69], or modality [49]. Only the number of ESCs 12–48 h after exercise was affected with larger effect sizes when percentage of male subjects was greater and when duration or load was higher. But given that only 6 out of 20 studies included females, and that the correlation to duration and load was lost when an ultra-marathon study was excluded [44], these relations may be questioned despite being significant. Certainly, an equal distribution of males and females should be achieved in future studies to clarify the influence of sex.

We thus conclude that the extent and the kinetics of the mobilization of stem and progenitor cells after exercise is susceptible to a variety of external and internal influences and moderators but none of them has a significant influence on its own. We therefore suggest that future studies standardize external influences as much as possible and report detailed information on subjects and their training status as well as all possible details of the interventions.

### Limitations

As a first limitation we need to point out that grouping stem and progenitor cells into enSCs, ESCs, HSCs and MSCs proved to be more challenging than assumed since no consensus exists on the definition of marker combinations defining these cell populations, i.e. subgroups using the same nomenclature (e.g. “hematopoietic” or “endothelial” progenitor cells) in different studies include cells defined by different markers or marker combinations. For example, Thjissen et al., 2006 [73] and Baker et al., 2017 [39] define “hematopoietic stem cells” as CD34^+^, while Agha et al., 2018 [37], Bonsignore et al., 2010 [41] and Adams et al., 2008 [36] include CD133^+^ cells in addition to CD34^+^ in their definition of “hematopoietic stem and progenitor cells” and Niemiro et al., 2017 [59] refer to CD34^+^ cells more generally as “circulating progenitor cells” while defining “hematopoietic stem and progenitor cells” as CD34^+^/CD45^dim^. This same definition problem was present also for endothelial and mesenchymal progenitors. Therefore, we decided to ignore the “group nomenclature” used in the original publications and instead extract the specific markers (or marker combinations) to identify cell subgroups. We are confident to thereby provide the best possible analysis of this data.

Second, studies contributing more than one effect size were corrected for by adjusting their individual weight, but the outcomes may still not be completely independent. This introduces a certain bias. However, an attempt for more detailed correction requires assumptions that may ultimately increase bias.

Third, the lack for correction for multiple testing may be perceived as a limitation. However, adjustment by the Bonferroni method [32], would have resulted in very large confidence intervals resulting in distorted representations of the true effect sizes. The statistical consultant therefore deemed the approach to be too conservative and not feasible in the present case.

Last, not all of the outcomes are equally well researched which limits the value of some of the analyses, e.g. for MSCs, where only 3 studies were available. However, we decided to still include all of the performed analyses in the study for the sake of completeness and to highlight weak points of present research, where further investigation is still needed.

## Conclusions

Acute exercise elicits an increase in circulating stem and progenitor cell numbers but the significance and extent, as well as the kinetics of this mobilization vary markedly between the different subgroups of stem cells. ESC numbers are elevated until up to 48 h after exercise, while HSCs and enSCs transiently increase immediately after exercise, dropping back to baseline shortly after. However, more studies on exercise-induced mobilization of MSCs are required, as this cell subgroup represents a particularly promising target regarding stem cell transplantation and therapy and its non-invasive mobilization could provide a valuable asset in the clinical setting.

Since the identification of a cell as hematopoietic, endothelial or mesenchymal progenitor by cell-marker-combinations is employed very heterogeneously, a consensus regarding cell surface markers defining respective stem and progenitor cell subgroups is essential in order to improve clarity of mechanisms and communication in future research.

## Data Availability

The datasets used and/or analyzed during the current study are available from the corresponding author on reasonable request.

## Abbreviations

AE: Aerobic Exercise
CFU: Colony-Forming-Unit
enSCs: Early and non-specified Stem and Progenitor Cells
ESCs: Endothelial Stem and Progenitor Cells
EU: European
f: female
Fig.: Figure
G-CSF: Granulocyte colony stimulating factor
HM: Half-Marathon
HSCs: Hematopoietic Stem and Progenitor Cells
HT: Hypoxic Training
LP: Late-Pubertal
M: Marathon
m: male
MeSH: Medical Subject Heading
MNCs: Mononuclear blood cells
MSCs: Mesenchymal Stem and Progenitor Cells
N or n: Number of Effect Sizes or Studies
NT: Normoxic Training
os: old sedentary
Paired diff: Paired mean difference
PRISMA: Preferred Reporting Items for Systematic Reviews and Meta-Analyses
r: Correlation Coefficient
RE: Resistance Exercise
s: sedentary
SA: South-Asian
SCs: Stem and Progenitor Cells
SD: Standard Deviation
SE: Standard Error
Std diff: Standardized difference
t: trained
ys: young sedentary
yt: young trained
