# Correction to: Changes in Circulating Stem and Progenitor Cell Numbers Following Acute Exercise in Healthy Human Subjects: a Systematic Review and Meta-Analysis

**DOI:** 10.1007/s12015-021-10161-7

**Published:** 2021-04-06

**Authors:** M. Schmid, J. M. Kröpfl, C. M. Spengler

**Affiliations:** 1 Exercise Physiology Lab, Institute of Human Movement Sciences and Sport ETH Zurich, Winterthurerstrasse 190, CH-8057 Zurich, Switzerland; 2 Zurich Center for Integrative Human Physiology (ZIHP), University of Zurich, Winterthurerstrasse 190, CH-8057 Zurich, Switzerland

**Correction to: Stem Cell Reviews and Reports.**

10.1007/s12015-020-10105-7

The article “Changes in Circulating Stem and Progenitor Cell Numbers Following Acute Exercise in Healthy Human Subjects: a Systematic Review and Meta-analysis”, written by M. Schmid, J. M. Kröpfl and C. M. Spengler, was originally published electronically on the publisher’s internet portal on 02 January 2021 without open access. With the author(s)’ decision to opt for Open Choice the copyright of the article changed on 18 January 2021 to © The Author(s) 2021 and the article is forthwith distributed under a Creative Commons Attribution 4.0 International License, which permits use, sharing, adaptation, distribution and reproduction in any medium or format, as long as you give appropriate credit to the original author(s) and the source, provide a link to the Creative Commons licence, and indicate if changes were made. The images or other third party material in this article are included in the article’s Creative Commons licence, unless indicated otherwise in a credit line to the material. If material is not included in the article’s Creative Commons licence and your intended use is not permitted by statutory regulation or exceeds the permitted use, you will need to obtain permission directly from the copyright holder. To view a copy of this licence, visit http://creativecommons.org/licenses/by/4.0/.

